# Supplementary material for: The Influence of Elastic Support of Component Glass Panes on Deflection and Stress in Insulating Glass Units—Analytical Model
Source: Materials (Basel). 2024 Sep 23;17(18):4669. doi: 10.3390/ma17184669 (PMC11433723; doi:10.3390/ma17184669)
Supplement: Supplementary file 1 [file materials-17-04669-s001.zip › File S1.pdf]

File S1
Coefficients for Calculating Supporting Moments.

$$(m_y)_{y=\pm b/2} = \sum_{i=1,3,5\ldots} (-1)^{(i-1)/2} \cdot F_i \cdot \cos\frac{i\pi x}{a} \qquad (m_x)_{x=\pm a/2} = \sum_{i=1,3,5\ldots} (-1)^{(i-1)/2} \cdot G_i \cdot \cos\frac{i\pi y}{b} \quad \text{(A1)}$$

$$F' = F'_1 - F'_3 + F'_5 - F'_7 + F'_9 \qquad G' = G'_1 - G'_3 + G'_5 - G'_7 + G'_9 \quad \text{(A2)}$$

$$(m_y)_{x=0,\ y=\pm b/2} = F' \cdot q \cdot a^2 \qquad (m_x)_{x=\pm a/2,\, y=0} = G' \cdot q \cdot a^2 \quad \text{(A3)}$$

For $R_c \rightarrow \infty$ **(simply support)** all coefficients equal 0.

Table S1-1. Coefficients for calculating supporting moments for R_c **= 100**

$\varepsilon = b/a$	F'_1	F'_3	F'_5	F'_7	F'_9	F'	G'_1	G'_3	G'_5	G'_7	G'_9	G'
**1.0**	-0.00084605	-0.00001519	-0.00000190	-0.00000047	-0.00000017	**-0.00083245**	-0.00084605	-0.00001519	-0.00000190	-0.00000047	-0.00000017	**-0.00083245**
**1.1**	-0.00092901	-0.00001512	-0.00000188	-0.00000047	-0.00000016	**-0.00091546**	-0.00100707	-0.00002021	-0.00000253	-0.00000063	-0.00000022	**-0.00098897**
**1.2**	-0.00099785	-0.00001506	-0.00000186	-0.00000046	-0.00000016	**-0.00098435**	-0.00116362	-0.00002621	-0.00000329	-0.00000082	-0.00000029	**-0.00114017**
**1.3**	-0.00105393	-0.00001500	-0.00000185	-0.00000046	-0.00000016	**-0.00104048**	-0.00131297	-0.00003323	-0.00000420	-0.00000105	-0.00000037	**-0.00128326**
**1.4**	-0.00109897	-0.00001496	-0.00000184	-0.00000045	-0.00000016	**-0.00108555**	-0.00145350	-0.00004132	-0.00000526	-0.00000132	-0.00000046	**-0.00141658**
**1.5**	-0.00113471	-0.00001492	-0.00000183	-0.00000045	-0.00000016	**-0.00112133**	-0.00158437	-0.00005049	-0.00000650	-0.00000163	-0.00000058	**-0.00153932**
**1.6**	-0.00116284	-0.00001489	-0.00000183	-0.00000045	-0.00000015	**-0.00114948**	-0.00170536	-0.00006072	-0.00000791	-0.00000199	-0.00000070	**-0.00165126**
**1.7**	-0.00118479	-0.00001487	-0.00000182	-0.00000045	-0.00000015	**-0.00117145**	-0.00181664	-0.00007199	-0.00000952	-0.00000240	-0.00000085	**-0.00175262**
**1.8**	-0.00120183	-0.00001485	-0.00000182	-0.00000045	-0.00000015	**-0.00118850**	-0.00191865	-0.00008426	-0.00001133	-0.00000287	-0.00000102	**-0.00184387**
**1.9**	-0.00121498	-0.00001484	-0.00000182	-0.00000045	-0.00000015	**-0.00120167**	-0.00201193	-0.00009746	-0.00001335	-0.00000339	-0.00000120	**-0.00192563**
**2.0**	-0.00122509	-0.00001482	-0.00000182	-0.00000045	-0.00000015	**-0.00121178**	-0.00209713	-0.00011153	-0.00001559	-0.00000397	-0.00000141	**-0.00199863**
**3.0**	-0.00125524	-0.00001479	-0.00000181	-0.00000044	-0.00000015	**-0.00124196**	-0.00263542	-0.00028116	-0.00005050	-0.00001369	-0.00000496	**-0.00239603**
**5.0**	-0.00125711	-0.00001481	-0.00000182	-0.00000045	-0.00000015	**-0.00124383**	-0.00299566	-0.00059341	-0.00016869	-0.00005697	-0.00002267	**-0.00253664**

Table S1-2. Coefficients for calculating supporting moments for R_c = **10**

$\varepsilon = b/a$	F'_1	F'_3	F'_5	F'_7	F'_9	F'	G'_1	G'_3	G'_5	G'_7	G'_9	G'
1.0	-0.00729667	-0.00009922	-0.00000643	0.00000000	0.00000060	**-0.00720327**	-0.00729667	-0.00009922	-0.00000643	0.00000000	0.00000060	**-0.00720327**
1.1	-0.00795601	-0.00009289	-0.00000509	0.00000048	0.00000083	**-0.00786786**	-0.00863289	-0.00013503	-0.00000909	-0.00000017	0.00000073	**-0.00850604**
1.2	-0.00848971	-0.00008758	-0.00000400	0.00000086	0.00000101	**-0.00840599**	-0.00991808	-0.00017980	-0.00001275	-0.00000053	0.00000082	**-0.00974969**
1.3	-0.00891283	-0.00008327	-0.00000313	0.00000117	0.00000115	**-0.00883272**	-0.01113086	-0.00023432	-0.00001762	-0.00000115	0.00000082	**-0.01091219**
1.4	-0.00924270	-0.00007984	-0.00000245	0.00000141	0.00000126	**-0.00916546**	-0.01225948	-0.00029912	-0.00002389	-0.00000208	0.00000074	**-0.01198143**
1.5	-0.00949637	-0.00007716	-0.00000192	0.00000160	0.00000135	**-0.00942139**	-0.01329937	-0.00037443	-0.00003170	-0.00000337	0.00000054	**-0.01295275**
1.6	-0.00968923	-0.00007509	-0.00000152	0.00000174	0.00000142	**-0.00961598**	-0.01425085	-0.00046019	-0.00004123	-0.00000506	0.00000021	**-0.01382662**
1.7	-0.00983444	-0.00007352	-0.00000122	0.00000185	0.00000147	**-0.00976252**	-0.01511735	-0.00055611	-0.00005258	-0.00000720	-0.00000027	**-0.01460689**
1.8	-0.00994286	-0.00007233	-0.00000099	0.00000193	0.00000150	**-0.00987194**	-0.01590413	-0.00066169	-0.00006588	-0.00000983	-0.00000091	**-0.01529940**
1.9	-0.01002320	-0.00007144	-0.00000082	0.00000199	0.00000153	**-0.00995303**	-0.01661732	-0.00077625	-0.00008122	-0.00001298	-0.00000173	**-0.01591104**
2.0	-0.01008232	-0.00007078	-0.00000069	0.00000203	0.00000155	**-0.01001272**	-0.01726338	-0.00089901	-0.00009868	-0.00001669	-0.00000274	**-0.01644909**
3.0	-0.01023072	-0.00006913	-0.00000041	0.00000212	0.00000159	**-0.01016254**	-0.02122337	-0.00237654	-0.00039434	-0.00009059	-0.00002592	**-0.01917650**
5.0	-0.01023621	-0.00007006	-0.00000079	0.00000195	0.00000150	**-0.01016739**	-0.02375523	-0.00493973	-0.00142586	-0.00046506	-0.00017220	**-0.01994851**

Table S1-3. Coefficients for calculating supporting moments for R_c = **5**

$\varepsilon = b/a$	F'_1	F'_3	F'_5	F'_7	F'_9	F'	G'_1	G'_3	G'_5	G'_7	G'_9	G'
**1.0**	-0.01265917	-0.00011533	0.00000683	0.00000741	0.00000475	**-0.01253967**	-0.01265917	-0.00011533	0.00000683	0.00000741	0.00000475	**-0.01253967**
**1.1**	-0.01372213	-0.00009512	0.00001101	0.00000890	0.00000544	**-0.01361945**	-0.01490359	-0.00016565	0.00000689	0.00000916	0.00000602	**-0.01473419**
**1.2**	-0.01456333	-0.00007859	0.00001435	0.00001008	0.00000599	**-0.01447448**	-0.01704327	-0.00023297	0.00000530	0.00001068	0.00000727	**-0.01680842**
**1.3**	-0.01521366	-0.00006547	0.00001695	0.00001100	0.00000642	**-0.01513583**	-0.01904423	-0.00031921	0.00000155	0.00001181	0.00000844	**-0.01872685**
**1.4**	-0.01570681	-0.00005529	0.00001893	0.00001170	0.00000675	**-0.01563755**	-0.02088963	-0.00042556	-0.00000478	0.00001243	0.00000949	**-0.02047180**
**1.5**	-0.01607469	-0.00004754	0.00002042	0.00001223	0.00000699	**-0.01601196**	-0.02257498	-0.00055248	-0.00001408	0.00001242	0.00001036	**-0.02203865**
**1.6**	-0.01634523	-0.00004173	0.00002153	0.00001262	0.00000717	**-0.01628742**	-0.02410396	-0.00069980	-0.00002667	0.00001167	0.00001101	**-0.02343149**
**1.7**	-0.01654167	-0.00003743	0.00002234	0.00001291	0.00000731	**-0.01648749**	-0.02548515	-0.00086677	-0.00004280	0.00001011	0.00001142	**-0.02465987**
**1.8**	-0.01668262	-0.00003429	0.00002293	0.00001312	0.00000741	**-0.01663110**	-0.02672971	-0.00105221	-0.00006270	0.00000764	0.00001155	**-0.02573630**
**1.9**	-0.01678260	-0.00003202	0.00002336	0.00001327	0.00000748	**-0.01673301**	-0.02784986	-0.00125460	-0.00008656	0.00000420	0.00001137	**-0.02667465**
**2.0**	-0.01685274	-0.00003039	0.00002366	0.00001338	0.00000753	**-0.01680453**	-0.02885784	-0.00147220	-0.00011452	-0.00000029	0.00001085	**-0.02748902**
**3.0**	-0.01699664	-0.00002677	0.00002435	0.00001363	0.00000765	**-0.01695150**	-0.03489296	-0.00407085	-0.00062583	-0.00011486	-0.00001817	**-0.03135126**
**5.0**	-0.01699960	-0.00002903	0.00002347	0.00001323	0.00000745	**-0.01695289**	-0.03862206	-0.00833457	-0.00244244	-0.00077269	-0.00026497	**-0.03222222**

Table S1-4. Coefficients for calculating supporting moments for R_c = 2

$\varepsilon = b/a$	F'_1	F'_3	F'_5	F'_7	F'_9	F'	G'_1	G'_3	G'_5	G'_7	G'_9	G'
**1.0**	-0.02264867	0.00005657	0.00009762	0.00004871	0.00002624	**-0.02263009**	-0.02264867	0.00005657	0.00009762	0.00004871	0.00002624	**-0.02263009**
**1.1**	-0.02427944	0.00012947	0.00011227	0.00005386	0.00002862	**-0.02432188**	-0.02642706	0.00001158	0.00011767	0.00006073	0.00003306	**-0.02634863**
**1.2**	-0.02550840	0.00018665	0.00012343	0.00005776	0.00003042	**-0.02559896**	-0.02997068	-0.00007313	0.00013443	0.00007257	0.00004008	**-0.02979562**
**1.3**	-0.02640694	0.00023000	0.00013169	0.00006065	0.00003177	**-0.02653413**	-0.03322912	-0.00020270	0.00014642	0.00008379	0.00004709	**-0.03291671**
**1.4**	-0.02704643	0.00026194	0.00013765	0.00006274	0.00003275	**-0.02720071**	-0.03618384	-0.00037966	0.00015242	0.00009399	0.00005393	**-0.03569182**
**1.5**	-0.02749032	0.00028490	0.00014187	0.00006423	0.00003345	**-0.02766413**	-0.03883801	-0.00060432	0.00015149	0.00010287	0.00006050	**-0.03812457**
**1.6**	-0.02779096	0.00030106	0.00014482	0.00006528	0.00003396	**-0.02797853**	-0.04120796	-0.00087513	0.00014290	0.00011018	0.00006668	**-0.04023342**
**1.7**	-0.02798944	0.00031220	0.00014684	0.00006602	0.00003432	**-0.02818650**	-0.04331691	-0.00118917	0.00012608	0.00011572	0.00007242	**-0.04204497**
**1.8**	-0.02811679	0.00031974	0.00014823	0.00006655	0.00003458	**-0.02832027**	-0.04519079	-0.00154252	0.00010060	0.00011931	0.00007762	**-0.04358937**
**1.9**	-0.02819578	0.00032475	0.00014918	0.00006693	0.00003478	**-0.02840351**	-0.04685562	-0.00193064	0.00006613	0.00012080	0.00008225	**-0.04489741**
**2.0**	-0.02824267	0.00032803	0.00014984	0.00006721	0.00003493	**-0.02845314**	-0.04833603	-0.00234863	0.00002242	0.00012004	0.00008623	**-0.04599878**
**3.0**	-0.02827377	0.00033508	0.00015246	0.00006861	0.00003574	**-0.02848925**	-0.05685154	-0.00717497	-0.00091978	-0.00004034	0.00007710	**-0.05047892**
**5.0**	-0.02826956	0.00033498	0.00015274	0.00006880	0.00003585	**-0.02848474**	-0.06183165	-0.01419975	-0.00430515	-0.00128062	-0.00035350	**-0.05100993**

Table S1-5. Coefficients for calculating supporting moments for R_c = 1

$\varepsilon = b/a$	F'_1	F'_3	F'_5	F'_7	F'_9	F'	G'_1	G'_3	G'_5	G'_7	G'_9	G'
**1.0**	-0.03074254	0.00053143	0.00028743	0.00013124	0.00006831	**-0.03104947**	-0.03074254	0.00053143	0.00028743	0.00013124	0.00006831	**-0.03104947**
**1.1**	-0.03265460	0.00068396	0.00031717	0.00014150	0.00007303	**-0.03308986**	-0.03562355	0.00052900	0.00034610	0.00016212	0.00008515	**-0.03588342**
**1.2**	-0.03402600	0.00079899	0.00033880	0.00014896	0.00007648	**-0.03455866**	-0.04014023	0.00045279	0.00039853	0.00019283	0.00010246	**-0.04028485**
**1.3**	-0.03497156	0.00088232	0.00035400	0.00015423	0.00007896	**-0.03557516**	-0.04423519	0.00029487	0.00044183	0.00022242	0.00011986	**-0.04419079**
**1.4**	-0.03559910	0.00094062	0.00036438	0.00015788	0.00008071	**-0.03625252**	-0.04789594	0.00005298	0.00047388	0.00025021	0.00013705	**-0.04758819**
**1.5**	-0.03599936	0.00098012	0.00037131	0.00016040	0.00008196	**-0.03668662**	-0.05113891	-0.00027067	0.00049319	0.00027565	0.00015384	**-0.05049687**
**1.6**	-0.03624331	0.00100609	0.00037588	0.00016216	0.00008287	**-0.03695281**	-0.05399654	-0.00067070	0.00049868	0.00029838	0.00017008	**-0.05295546**
**1.7**	-0.03638362	0.00102267	0.00037889	0.00016341	0.00008356	**-0.03710725**	-0.05650829	-0.00113960	0.00048964	0.00031806	0.00018564	**-0.05501147**
**1.8**	-0.03645776	0.00103296	0.00038093	0.00016436	0.00008412	**-0.03719004**	-0.05871492	-0.00166864	0.00046553	0.00033444	0.00020042	**-0.05671477**
**1.9**	-0.03649143	0.00103921	0.00038239	0.00016514	0.00008459	**-0.03722879**	-0.06065533	-0.00224849	0.00042600	0.00034725	0.00021431	**-0.05811376**
**2.0**	-0.03650162	0.00104296	0.00038353	0.00016582	0.00008502	**-0.03724185**	-0.06236492	-0.00286977	0.00037082	0.00035624	0.00022722	**-0.05925334**
**3.0**	-0.03643410	0.00105820	0.00039399	0.00017211	0.00008882	**-0.03718160**	-0.07191881	-0.00969662	-0.00100320	0.00018663	0.00027541	**-0.06313661**
**5.0**	-0.03640675	0.00108315	0.00040600	0.00017799	0.00009200	**-0.03716990**	-0.07730570	-0.01856582	-0.00581978	-0.00163791	-0.00032431	**-0.06324605**

Table S1-6. Coefficients for calculating supporting moments for R_c = 0.5

$\varepsilon = b/a$	F'_1	F'_3	F'_5	F'_7	F'_9	F'	G'_1	G'_3	G'_5	G'_7	G'_9	G'
**1.0**	-0.03744749	0.00137624	0.00061689	0.00027448	0.00014120	**-0.03834013**	-0.03744749	0.00137624	0.00061689	0.00027448	0.00014120	**-0.03834013**
**1.1**	-0.03946888	0.00163630	0.00066592	0.00029104	0.00014878	**-0.04058152**	-0.04315456	0.00143816	0.00073489	0.00033497	0.00017384	**-0.04401897**
**1.2**	-0.04084630	0.00182484	0.00069989	0.00030260	0.00015420	**-0.04211964**	-0.04837450	0.00137550	0.00084080	0.00039485	0.00020724	**-0.04909681**
**1.3**	-0.04173785	0.00195527	0.00072255	0.00031051	0.00015806	**-0.04312302**	-0.05304841	0.00118030	0.00093008	0.00045260	0.00024078	**-0.05351045**
**1.4**	-0.04228424	0.00204174	0.00073725	0.00031592	0.00016086	**-0.04374378**	-0.05717442	0.00085452	0.00099975	0.00050713	0.00027404	**-0.05726228**
**1.5**	-0.04259789	0.00209681	0.00074667	0.00031973	0.00016299	**-0.04410477**	-0.06078526	0.00040692	0.00104797	0.00055770	0.00030671	**-0.06039520**
**1.6**	-0.04276224	0.00213052	0.00075280	0.00032258	0.00016472	**-0.04429782**	-0.06393091	-0.00014957	0.00107362	0.00060376	0.00033858	**-0.06297291**
**1.7**	-0.04283568	0.00215041	0.00075702	0.00032492	0.00016624	**-0.04438775**	-0.06666710	-0.00079986	0.00107608	0.00064489	0.00036946	**-0.06506659**
**1.8**	-0.04285717	0.00216181	0.00076026	0.00032702	0.00016767	**-0.04441808**	-0.06904850	-0.00152812	0.00105496	0.00068070	0.00039919	**-0.06674694**
**1.9**	-0.04285147	0.00216837	0.00076310	0.00032906	0.00016907	**-0.04441673**	-0.07112532	-0.00231865	0.00101007	0.00071080	0.00042757	**-0.06807983**
**2.0**	-0.04283355	0.00217243	0.00076588	0.00033112	0.00017047	**-0.04440075**	-0.07294187	-0.00315656	0.00094139	0.00073482	0.00045443	**-0.06912431**
**3.0**	-0.04271004	0.00221822	0.00080297	0.00035425	0.00018471	**-0.04429483**	-0.08288343	-0.01182615	-0.00093181	0.00056654	0.00059255	**-0.07196308**
**5.0**	-0.04261560	0.00232615	0.00085643	0.00038102	0.00019943	**-0.04426692**	-0.08835261	-0.02194697	-0.00710404	-0.00190397	-0.00020466	**-0.07181037**

Table S1-7. Coefficients for calculating supporting moments for R_c = 0.1

$\varepsilon = b/a$	F'_1	F'_3	F'_5	F'_7	F'_9	F'	G'_1	G'_3	G'_5	G'_7	G'_9	G'
**1.0**	-0.04541826	0.00359323	0.00158179	0.00071801	0.00037308	**-0.04777463**	-0.04541826	0.00359323	0.00158179	0.00071801	0.00037308	**-0.04777463**
**1.1**	-0.04743192	0.00407592	0.00166830	0.00074647	0.00038675	**-0.05019925**	-0.05200204	0.00373066	0.00183055	0.00085177	0.00044652	**-0.05430740**
**1.2**	-0.04870305	0.00440618	0.00172463	0.00076648	0.00039757	**-0.05175351**	-0.05793366	0.00362896	0.00204687	0.00098193	0.00052108	**-0.05997660**
**1.3**	-0.04944834	0.00461998	0.00176069	0.00078137	0.00040685	**-0.05268215**	-0.06315905	0.00329363	0.00222416	0.00110564	0.00059555	**-0.06473860**
**1.4**	-0.04984708	0.00475167	0.00178434	0.00079359	0.00041552	**-0.05319249**	-0.06769756	0.00274613	0.00235929	0.00122108	0.00066905	**-0.06863643**
**1.5**	-0.05003234	0.00482934	0.00180125	0.00080479	0.00042414	**-0.05344108**	-0.07160889	0.00201602	0.00245126	0.00132712	0.00074095	**-0.07175982**
**1.6**	-0.05009527	0.00487383	0.00181518	0.00081596	0.00043299	**-0.05353688**	-0.07496912	0.00113552	0.00250022	0.00142298	0.00081076	**-0.07421664**
**1.7**	-0.05009424	0.00489959	0.00182844	0.00082762	0.00044222	**-0.05355078**	-0.07785620	0.00013628	0.00250681	0.00150808	0.00087806	**-0.07611570**
**1.8**	-0.05006428	0.00491600	0.00184232	0.00084002	0.00045184	**-0.05352614**	-0.08034237	-0.00095230	0.00247188	0.00158187	0.00094243	**-0.07755764**
**1.9**	-0.05002494	0.00492885	0.00185746	0.00085318	0.00046179	**-0.05348772**	-0.08249106	-0.00210385	0.00239640	0.00164385	0.00100349	**-0.07863117**
**2.0**	-0.04998606	0.00494147	0.00187408	0.00086704	0.00047201	**-0.05344849**	-0.08435625	-0.00329531	0.00228147	0.00169347	0.00106081	**-0.07941213**
**3.0**	-0.04979198	0.00517940	0.00209087	0.00101713	0.00057219	**-0.05332545**	-0.09436302	-0.01444182	-0.00062395	0.00143521	0.00135040	**-0.08062995**
**5.0**	-0.04943159	0.00570647	0.00240076	0.00119193	0.00067701	**-0.05325221**	-0.09974710	-0.02570620	-0.00870885	-0.00222115	0.00008088	**-0.08044773**

Table S1-8. Coefficients for calculating supporting moments for R_c **= 0 (clamped fixity)**

ε	F'_1	F'_3	F'_5	F'_7	F'_9	F'	G'_1	G'_3	G'_5	G'_7	G'_9	G'
**1.0**	-0.04800109	0.00489427	0.00227302	0.00107940	0.00057976	**-0.05112198**	-0.04800109	0.00489427	0.00227302	0.00107940	0.00057976	**-0.05112198**
**1.1**	-0.04998938	0.00549271	0.00238122	0.00111730	0.00060118	**-0.05361700**	-0.05483568	0.00501544	0.00257594	0.00124985	0.00067456	**-0.05785047**
**1.2**	-0.05121031	0.00589466	0.00245219	0.00114701	0.00062156	**-0.05517823**	-0.06096181	0.00484325	0.00283229	0.00141382	0.00077076	**-0.06361582**
**1.3**	-0.05190009	0.00615025	0.00249985	0.00117296	0.00064239	**-0.05608107**	-0.06632895	0.00439521	0.00303552	0.00156760	0.00086641	**-0.06838983**
**1.4**	-0.05224919	0.00630565	0.00253469	0.00119807	0.00066441	**-0.05655382**	-0.07096557	0.00370578	0.00318349	0.00170901	0.00096014	**-0.07223673**
**1.5**	-0.05239522	0.00639749	0.00256388	0.00122409	0.00068791	**-0.05676500**	-0.07494174	0.00281617	0.00327661	0.00183680	0.00105097	**-0.07526713**
**1.6**	-0.05242968	0.00645215	0.00259188	0.00125183	0.00071285	**-0.05682893**	-0.07834286	0.00176797	0.00331640	0.00195029	0.00113821	**-0.07760652**
**1.7**	-0.05240927	0.00648733	0.00262119	0.00128157	0.00073903	**-0.05681794**	-0.08125430	0.00059998	0.00330476	0.00204901	0.00122130	**-0.07937722**
**1.8**	-0.05236665	0.00651414	0.00265304	0.00131324	0.00076619	**-0.05677480**	-0.08375375	-0.00065327	0.00324360	0.00213254	0.00129977	**-0.08068964**
**1.9**	-0.05231908	0.00653913	0.00268785	0.00134658	0.00079403	**-0.05672291**	-0.08590851	-0.00196189	0.00313485	0.00220051	0.00137318	**-0.08163910**
**2.0**	-0.05227451	0.00656587	0.00272559	0.00138126	0.00082226	**-0.05667378**	-0.08777516	-0.00330055	0.00298046	0.00225251	0.00144108	**-0.08230558**
**3.0**	-0.05201711	0.00701635	0.00318148	0.00173552	0.00108558	**-0.05650192**	-0.09774028	-0.01532291	-0.00048291	0.00185427	0.00174322	**-0.08301133**
**5.0**	-0.05146443	0.00792314	0.00379206	0.00212497	0.00134633	**-0.05637414**	-0.10307072	-0.02686857	-0.00926854	-0.00235761	0.00018565	**-0.08292742**
