# Supplementary material for: The Influence of Elastic Support of Component Glass Panes on Deflection and Stress in Insulating Glass Units—Analytical Model
Source: Materials (Basel). 2024 Sep 23;17(18):4669. doi: 10.3390/ma17184669 (PMC11433723; doi:10.3390/ma17184669)
Supplement: Supplementary file 1 [file materials-17-04669-s001.zip › File S2.pdf]

File S2
Coefficients for Calculating Deflection and Change in Cavity Volume in IGUs.

Table S2-1. Coefficients α'_w used to calculate the deflection in the glass pane center.

$\varepsilon = b/a$	Index $R_c = 2\pi \cdot D/(a \cdot C)$								
	Simply support	100	10	5	2	1	0.5	0.1	Clamped fixity
1.0	0.00406241	0.00401321	0.00363803	0.00332608	0.00274479	0.00227349	0.00188264	0.00141686	0.00126538
1.1	0.00486901	0.00480711	0.00433851	0.00395346	0.00324670	0.00268366	0.00222329	0.00168225	0.00150813
1.2	0.00565059	0.00557528	0.00500905	0.00454874	0.00371524	0.00306148	0.00253346	0.00192039	0.00172493
1.3	0.00639227	0.00630313	0.00563708	0.00510093	0.00414195	0.00340014	0.00280753	0.00212685	0.00191169
1.4	0.00708497	0.00698184	0.00621559	0.00560437	0.00452323	0.00369741	0.00304415	0.00230117	0.00206821
1.5	0.00772408	0.00760699	0.00674171	0.00605727	0.00485896	0.00395411	0.00324476	0.00244536	0.00219659
1.6	0.00830817	0.00817737	0.00721548	0.00646053	0.00515117	0.00417288	0.00341236	0.00256260	0.00230003
1.7	0.00883806	0.00869392	0.00763881	0.00681665	0.00540313	0.00435733	0.00355063	0.00265652	0.00238209
1.8	0.00931597	0.00915898	0.00801473	0.00712910	0.00561871	0.00451139	0.00366344	0.00273071	0.00244623
1.9	0.00974501	0.00957574	0.00834691	0.00740176	0.00580194	0.00463900	0.00375452	0.00278852	0.00249561
2.0	0.01012872	0.00994779	0.00863922	0.00763861	0.00595675	0.00474386	0.00382728	0.00283290	0.00253303
3.0	0.01223287	0.01197022	0.01012056	0.00876417	0.00659504	0.00511680	0.00404880	0.00294066	0.00261743
5.0	0.01297089	0.01265996	0.01051828	0.00899996	0.00665935	0.00512151	0.00403573	0.00292681	0.00260568

Table S2-2. Coefficients α'_v used to calculate the change in cavity volume (own research).

$\varepsilon = b/a$	Index $R_c = 2\pi \cdot D/(a \cdot C)$								
	Simply support	100	10	5	2	1	0.5	0.1	Clamped fixity
1.0	0.00170250	0.00167930	0.00150240	0.00135537	0.00108159	0.00085993	0.00067656	0.00045922	0.00038912
1.1	0.00224595	0.00221385	0.00197093	0.00177140	0.00140548	0.00111444	0.00087711	0.00059977	0.00051124
1.2	0.00284803	0.00280548	0.00248566	0.00222581	0.00175577	0.00138783	0.00109156	0.00074966	0.00064154
1.3	0.00349885	0.00344439	0.00303753	0.00271025	0.00212560	0.00167445	0.00131529	0.00090539	0.00077684
1.4	0.00418939	0.00412165	0.00361855	0.00321757	0.00250937	0.00196991	0.00154479	0.00106442	0.00091490
1.5	0.00491177	0.00482953	0.00422202	0.00374192	0.00290275	0.00227097	0.00177763	0.00122512	0.00105429
1.6	0.00565937	0.00556155	0.00484251	0.00427871	0.00330255	0.00257540	0.00201223	0.00138651	0.00119420
1.7	0.00642676	0.00631241	0.00547577	0.00482446	0.00370651	0.00288170	0.00224760	0.00154806	0.00133416
1.8	0.00720956	0.00707787	0.00611848	0.00537655	0.00411305	0.00318894	0.00248320	0.00170949	0.00147400
1.9	0.00800429	0.00785458	0.00676815	0.00593307	0.00452114	0.00349657	0.00271873	0.00187071	0.00161364
2.0	0.00880821	0.00863990	0.00742289	0.00649266	0.00493010	0.00380426	0.00295408	0.00203172	0.00175309
3.0	0.01705529	0.01668480	0.01406769	0.01213768	0.00902410	0.00687420	0.00529944	0.00363642	0.00314329
5.0	0.03371665	0.03292410	0.02741377	0.02344549	0.01720473	0.01300561	0.00998443	0.00684346	0.00592211
