# Supplementary material for: The Influence of Elastic Support of Component Glass Panes on Deflection and Stress in Insulating Glass Units—Analytical Model
Source: Materials (Basel). 2024 Sep 23;17(18):4669. doi: 10.3390/ma17184669 (PMC11433723; doi:10.3390/ma17184669)
Supplement: Supplementary file 1 [file materials-17-04669-s001.zip › File S3.pdf]

File S3
Coefficients for Calculating Stress in Glass Panes.

Stress from supporting moments

$$\left(\sigma_{\mathrm{x,ed}}\right)_{x=\pm a/2,\, y=0} = k_{\mathrm{x,ed}} \cdot \frac{6 \cdot q \cdot a^2}{d^2} \qquad \left(\sigma_{\mathrm{y,ed}}\right)_{x=0,\, y=\pm b/2} = k_{\mathrm{y,ed}} \cdot \frac{6 \cdot q \cdot a^2}{d^2} \tag{C1}$$

Stress in the glass pane center

$$\left(\sigma_{\mathrm{x,cen}}\right)_{x=0,\, y=0} = k_{\mathrm{x,cen}} \cdot \frac{6 \cdot q \cdot a^2}{d^2} \qquad \left(\sigma_{\mathrm{y,ed}}\right)_{x=0,\, y=0} = k_{\mathrm{y,cen}} \cdot \frac{6 \cdot q \cdot a^2}{d^2} \tag{C2}$$

Table S3-1. Coefficients for calculating stress in IGUs for $R_c \to \infty$ **(simply support)**

$\varepsilon = b/a$	$\mu = 0.2$		$\mu = 0.23$	
	$k_{x,cen}$	$k_{y,cen}$	$k_{x,cen}$	$k_{y,cen}$
**1.0**	**0.04426385**	**0.04421502**	**0.04536892**	**0.04532192**
**1.1**	**0.05195518**	**0.04485843**	**0.05303229**	**0.04620166**
**1.2**	**0.05930589**	**0.04485606**	**0.06033698**	**0.04642902**
**1.3**	**0.06620238**	**0.04438420**	**0.06717562**	**0.04617562**
**1.4**	**0.07258194**	**0.04358741**	**0.07349041**	**0.04558318**
**1.5**	**0.07841951**	**0.04257939**	**0.07926000**	**0.04476388**
**1.6**	**0.08371643**	**0.04144679**	**0.08448841**	**0.04380389**
**1.7**	**0.08849134**	**0.04025401**	**0.08919620**	**0.04276778**
**1.8**	**0.09277354**	**0.03904776**	**0.09341395**	**0.04170289**
**1.9**	**0.09659817**	**0.03786108**	**0.09717759**	**0.04064314**
**2.0**	**0.10000287**	**0.03671657**	**0.10052524**	**0.03961218**
**3.0**	**0.11837558**	**0.02891654**	**0.11853937**	**0.03243505**
**5.0**	**0.12464621**	**0.02530684**	**0.12465801**	**0.02904387**

Table S3-2. Coefficients for calculating stress in IGUs for $R_c = 100$

$\varepsilon = b/a$	$\mu = 0.2$		$\mu = 0.23$	
	$k_{x,cen}$	$k_{y,cen}$	$k_{x,cen}$	$k_{y,cen}$
**1.0**	**0.04385955**	**0.04381072**	**0.04495451**	**0.04490752**
**1.1**	**0.05145934**	**0.04442309**	**0.05252594**	**0.04575355**
**1.2**	**0.05871228**	**0.04439260**	**0.05973259**	**0.04594991**
**1.3**	**0.06550645**	**0.04389536**	**0.06646877**	**0.04566809**
**1.4**	**0.07178090**	**0.04307590**	**0.07267840**	**0.04504983**
**1.5**	**0.07751231**	**0.04204789**	**0.07834185**	**0.04420735**
**1.6**	**0.08270352**	**0.04089797**	**0.08346468**	**0.04322684**
**1.7**	**0.08737450**	**0.03969045**	**0.08806874**	**0.04217284**
**1.8**	**0.09155562**	**0.03847193**	**0.09218565**	**0.04109259**
**1.9**	**0.09528280**	**0.03727528**	**0.09585213**	**0.04001990**
**2.0**	**0.09859427**	**0.03612294**	**0.09910690**	**0.03897824**
**3.0**	**0.11629736**	**0.02832055**	**0.11645552**	**0.03177784**
**5.0**	**0.12216201**	**0.02477834**	**0.12217282**	**0.02844104**

Table S3-3. Coefficients for calculating stress in IGUs for R_c = 10

$\varepsilon = b/a$	$\mu = 0.2$		$\mu = 0.23$	
	$k_{x,cen}$	$k_{y,cen}$	$k_{x,cen}$	$k_{y,cen}$
1.0	0.04077639	0.04072756	0.04179427	0.04174727
1.1	0.04770672	0.04112601	0.04869374	0.04235981
1.2	0.05425069	0.04090449	0.05518989	0.04234417
1.3	0.06030864	0.04023753	0.06118914	0.04187069
1.4	0.06583243	0.03926917	0.06664813	0.04108100
1.5	0.07081093	0.03811282	0.07155939	0.04008745
1.6	0.07525778	0.03685464	0.07593913	0.03897611
1.7	0.07920188	0.03555818	0.07981806	0.03781100
1.8	0.08268057	0.03426894	0.08323472	0.03663852
1.9	0.08573498	0.03301843	0.08623096	0.03549129
2.0	0.08840692	0.03182761	0.08884899	0.03439141
3.0	0.10165402	0.02415471	0.10177352	0.02718043
5.0	0.10504598	0.02116898	0.10505097	0.02431936

Table S3-4. Coefficients for calculating stress in IGUs for R_c = 5

$\varepsilon = b/a$	$\mu = 0.2$		$\mu = 0.23$	
	$k_{x,cen}$	$k_{y,cen}$	$k_{x,cen}$	$k_{y,cen}$
1.0	0.03821216	0.03816334	0.03916594	0.03911895
1.1	0.04462415	0.03841376	0.04554568	0.03956818
1.2	0.05062633	0.03806331	0.05149939	0.03940749
1.3	0.05612842	0.03728510	0.05694277	0.03880608
1.4	0.06109195	0.03622264	0.06184208	0.03790537
1.5	0.06551480	0.03498900	0.06619874	0.03681766
1.6	0.06941829	0.03366980	0.07003661	0.03562869
1.7	0.07283748	0.03232773	0.07339248	0.03440185
1.8	0.07581430	0.03100716	0.07630943	0.03318256
1.9	0.07839306	0.02973830	0.07883243	0.03200222
2.0	0.08061757	0.02854068	0.08100561	0.03088160
3.0	0.09090530	0.02114317	0.09099787	0.02385182
5.0	0.09290616	0.01864576	0.09290818	0.02143254

Table S3-5. Coefficients for calculating stress in IGUs for R_c = **2**

$\varepsilon = b/a$	$k_{x,ed}$	$k_{y,ed}$	$\mu = 0.2$		$\mu = 0.23$	
			$k_{x,cen}$	$k_{y,cen}$	$k_{x,cen}$	$k_{y,cen}$
**1.0**	**-0.00720327**	**-0.00720327**	**0.03343112**	**0.03338229**	**0.03426537**	**0.03421838**
**1.1**	**-0.00850604**	**-0.00786786**	**0.03896939**	**0.03342501**	**0.03977037**	**0.03443390**
**1.2**	**-0.00974969**	**-0.00840599**	**0.04407195**	**0.03289974**	**0.04482462**	**0.03407136**
**1.3**	**-0.01091219**	**-0.00883272**	**0.04866350**	**0.03197760**	**0.04935865**	**0.03329847**
**1.4**	**-0.01198143**	**-0.00916546**	**0.05272109**	**0.03080177**	**0.05335413**	**0.03225680**
**1.5**	**-0.01295275**	**-0.00942139**	**0.05625683**	**0.02948494**	**0.05682662**	**0.03105868**
**1.6**	**-0.01382662**	**-0.00961598**	**0.05930387**	**0.02811160**	**0.05981171**	**0.02978915**
**1.7**	**-0.01460689**	**-0.00976252**	**0.06190629**	**0.02674256**	**0.06235508**	**0.02850999**
**1.8**	**-0.01529940**	**-0.00987194**	**0.06411231**	**0.02541967**	**0.06450597**	**0.02726431**
**1.9**	**-0.01591104**	**-0.00995303**	**0.06597013**	**0.02417027**	**0.06631314**	**0.02608077**
**2.0**	**-0.01644909**	**-0.01001272**	**0.06752550**	**0.02301080**	**0.06782255**	**0.02497715**
**3.0**	**-0.01917650**	**-0.01016254**	**0.07368899**	**0.01643771**	**0.07374212**	**0.01863776**
**5.0**	**-0.01994851**	**-0.01016739**	**0.07418183**	**0.01482034**	**0.07418133**	**0.01704590**

Table S3-6. Coefficients for calculating stress in IGUs for R_c = **1**

$\varepsilon = b/a$	$k_{x,ed}$	$k_{y,ed}$	$\mu = 0.2$		$\mu = 0.23$	
			$k_{x,cen}$	$k_{y,cen}$	$k_{x,cen}$	$k_{y,cen}$
**1.0**	**-0.02263009**	**-0.02263009**	**0.02955021**	**0.02950139**	**0.03028744**	**0.03024045**
**1.1**	**-0.02634863**	**-0.02432188**	**0.03446881**	**0.02943497**	**0.03517323**	**0.03032815**
**1.2**	**-0.02979562**	**-0.02559896**	**0.03894250**	**0.02882229**	**0.03959981**	**0.02985911**
**1.3**	**-0.03291671**	**-0.02653413**	**0.04290554**	**0.02783502**	**0.04350722**	**0.02900185**
**1.4**	**-0.03569182**	**-0.02720071**	**0.04634555**	**0.02661761**	**0.04688769**	**0.02789955**
**1.5**	**-0.03812457**	**-0.02766413**	**0.04928442**	**0.02528313**	**0.04976649**	**0.02666525**
**1.6**	**-0.04023342**	**-0.02797853**	**0.05176324**	**0.02391526**	**0.05218707**	**0.02538339**
**1.7**	**-0.04204497**	**-0.02818650**	**0.05383178**	**0.02257290**	**0.05420073**	**0.02411406**
**1.8**	**-0.04358937**	**-0.02832027**	**0.05554188**	**0.02129532**	**0.05586022**	**0.02289791**
**1.9**	**-0.04489741**	**-0.02840351**	**0.05694355**	**0.02010690**	**0.05721599**	**0.02176072**
**2.0**	**-0.04599878**	**-0.02845314**	**0.05808293**	**0.01902103**	**0.05831432**	**0.02071724**
**3.0**	**-0.05047892**	**-0.02848925**	**0.06192749**	**0.01334810**	**0.06195757**	**0.01519991**
**5.0**	**-0.05100993**	**-0.02848474**	**0.06187251**	**0.01235191**	**0.06187180**	**0.01420822**

Table S3-7. Coefficients for calculating stress in IGUs for R_c = 0.5

$\varepsilon = b/a$	$k_{x,ed}$	$k_{y,ed}$	μ = 0.2		μ = 0.23	
			$k_{x,cen}$	$k_{y,cen}$	$k_{x,cen}$	$k_{y,cen}$
**1.0**	**-0.03834013**	**-0.03834013**	**0.02632570**	**0.02627687**	**0.02698231**	**0.02693532**
**1.1**	**-0.04401897**	**-0.04058152**	**0.03079269**	**0.02615405**	**0.03141755**	**0.02695286**
**1.2**	**-0.04909681**	**-0.04211964**	**0.03481118**	**0.02549888**	**0.03539045**	**0.02642736**
**1.3**	**-0.05351045**	**-0.04312302**	**0.03832173**	**0.02448641**	**0.03884742**	**0.02553093**
**1.4**	**-0.05726228**	**-0.04374378**	**0.04131999**	**0.02326387**	**0.04178873**	**0.02440972**
**1.5**	**-0.06039520**	**-0.04410477**	**0.04383548**	**0.02194524**	**0.04424730**	**0.02317794**
**1.6**	**-0.06297291**	**-0.04429782**	**0.04591540**	**0.02061330**	**0.04627260**	**0.02191932**
**1.7**	**-0.06506659**	**-0.04438775**	**0.04761374**	**0.01932475**	**0.04792005**	**0.02069190**
**1.8**	**-0.06674694**	**-0.04441808**	**0.04898473**	**0.01811594**	**0.04924470**	**0.01953349**
**1.9**	**-0.06807983**	**-0.04441673**	**0.05007932**	**0.01700804**	**0.05029783**	**0.01846672**
**2.0**	**-0.06912431**	**-0.04440075**	**0.05094344**	**0.01601123**	**0.05112540**	**0.01750314**
**3.0**	**-0.07196308**	**-0.04429483**	**0.05340726**	**0.01120812**	**0.05342372**	**0.01280705**
**5.0**	**-0.07181037**	**-0.04426692**	**0.05317762**	**0.01063090**	**0.05317748**	**0.01222626**

Table S3-8. Coefficients for calculating stress in IGUs for R_c = 0.1

$\varepsilon = b/a$	$k_{x,ed}$	$k_{y,ed}$	μ = 0.2		μ = 0.23	
			$k_{x,cen}$	$k_{y,cen}$	$k_{x,cen}$	$k_{y,cen}$
**1.0**	**-0.04777463**	**-0.04777463**	**0.02246681**	**0.02241798**	**0.02302695**	**0.02297996**
**1.1**	**-0.05430740**	**-0.05019925**	**0.02647571**	**0.02225854**	**0.02700581**	**0.02294679**
**1.2**	**-0.05997660**	**-0.05175351**	**0.03003167**	**0.02158074**	**0.03051837**	**0.02238435**
**1.3**	**-0.06473860**	**-0.05268215**	**0.03308143**	**0.02056882**	**0.03351744**	**0.02147406**
**1.4**	**-0.06863643**	**-0.05319249**	**0.03563042**	**0.01937536**	**0.03601321**	**0.02036771**
**1.5**	**-0.07175982**	**-0.05344108**	**0.03771786**	**0.01811517**	**0.03804822**	**0.01918064**
**1.6**	**-0.07421664**	**-0.05353688**	**0.03939852**	**0.01686847**	**0.03967942**	**0.01799425**
**1.7**	**-0.07611570**	**-0.05355078**	**0.04073134**	**0.01568740**	**0.04096700**	**0.01686221**
**1.8**	**-0.07755764**	**-0.05352614**	**0.04177312**	**0.01460293**	**0.04196837**	**0.01581707**
**1.9**	**-0.07863117**	**-0.05348772**	**0.04257540**	**0.01363080**	**0.04273526**	**0.01487609**
**2.0**	**-0.07941213**	**-0.05344849**	**0.04318335**	**0.01277619**	**0.04331271**	**0.01404582**
**3.0**	**-0.08062995**	**-0.05332545**	**0.04453610**	**0.00911289**	**0.04454253**	**0.01044769**
**5.0**	**-0.08044773**	**-0.05325221**	**0.04429256**	**0.00889277**	**0.04429363**	**0.01022133**

Table S3-9. Coefficients for calculating stress in IGUs for R_c = 0 **(clamped fixity)**

$\varepsilon = b/a$	$k_{x,ed}$	$k_{y,ed}$	$\mu = 0.2$		$\mu = 0.23$	
			$k_{x,cen}$	$k_{y,cen}$	$k_{x,cen}$	$k_{y,cen}$
**1.0**	**-0.05112198**	**-0.05112198**	**0.02120417**	**0.02115534**	**0.02173275**	**0.02168575**
**1.1**	**-0.05785047**	**-0.05361700**	**0.02508502**	**0.02099016**	**0.02558418**	**0.02164287**
**1.2**	**-0.06361582**	**-0.05517823**	**0.02851077**	**0.02031197**	**0.02896733**	**0.02107598**
**1.3**	**-0.06838983**	**-0.05608107**	**0.03142986**	**0.01930919**	**0.03183684**	**0.02017069**
**1.4**	**-0.07223673**	**-0.05655382**	**0.03385121**	**0.01813618**	**0.03420639**	**0.01908068**
**1.5**	**-0.07526713**	**-0.05676500**	**0.03581747**	**0.01690745**	**0.03612197**	**0.01792107**
**1.6**	**-0.07760652**	**-0.05682893**	**0.03738608**	**0.01570152**	**0.03764309**	**0.01677170**
**1.7**	**-0.07937722**	**-0.05681794**	**0.03861764**	**0.01456824**	**0.03883154**	**0.01568399**
**1.8**	**-0.08068964**	**-0.05677480**	**0.03956970**	**0.01353619**	**0.03974540**	**0.01468815**
**1.9**	**-0.08163910**	**-0.05672291**	**0.04029392**	**0.01261886**	**0.04043642**	**0.01379918**
**2.0**	**-0.08230558**	**-0.05667378**	**0.04083506**	**0.01181948**	**0.04094920**	**0.01302171**
**3.0**	**-0.08301133**	**-0.05650192**	**0.04194068**	**0.00853475**	**0.04194526**	**0.00979205**
**5.0**	**-0.08292742**	**-0.05637414**	**0.04171856**	**0.00839235**	**0.04172008**	**0.00964360**
