# Supplementary material for: The Influence of Elastic Support of Component Glass Panes on Deflection and Stress in Insulating Glass Units—Analytical Model
Source: Materials (Basel). 2024 Sep 23;17(18):4669. doi: 10.3390/ma17184669 (PMC11433723; doi:10.3390/ma17184669)
Supplement: Supplementary file 1 [file materials-17-04669-s001.zip › File S4.pdf]

# File S4
# Sample Maps of Static Quantities.

Figures show deflection and stress maps for the plate with reference parameters at $C$ = 1 **kNm/(m·rad)**.

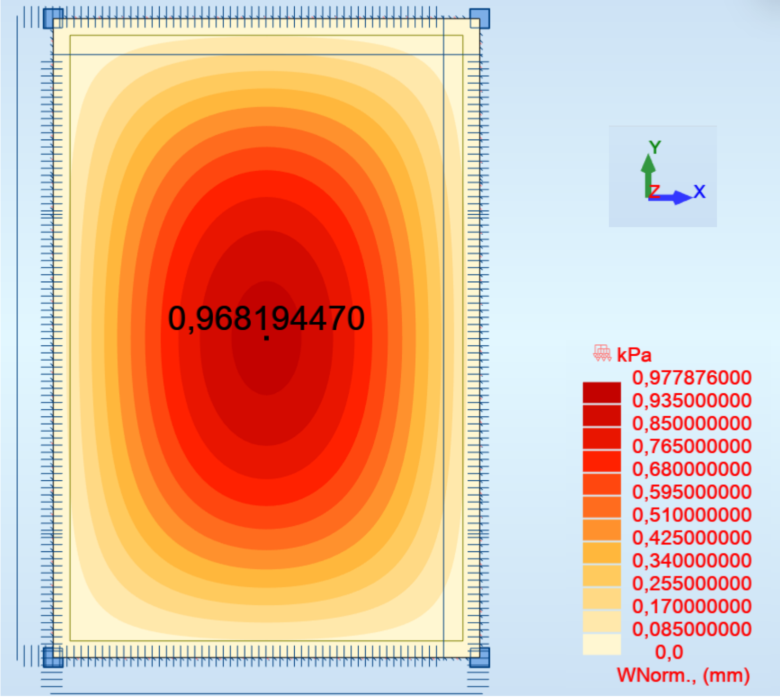


**Figure S4-1.** Map of deflection for the plate with reference parameters at $C$ = 1 kNm/(m·rad).

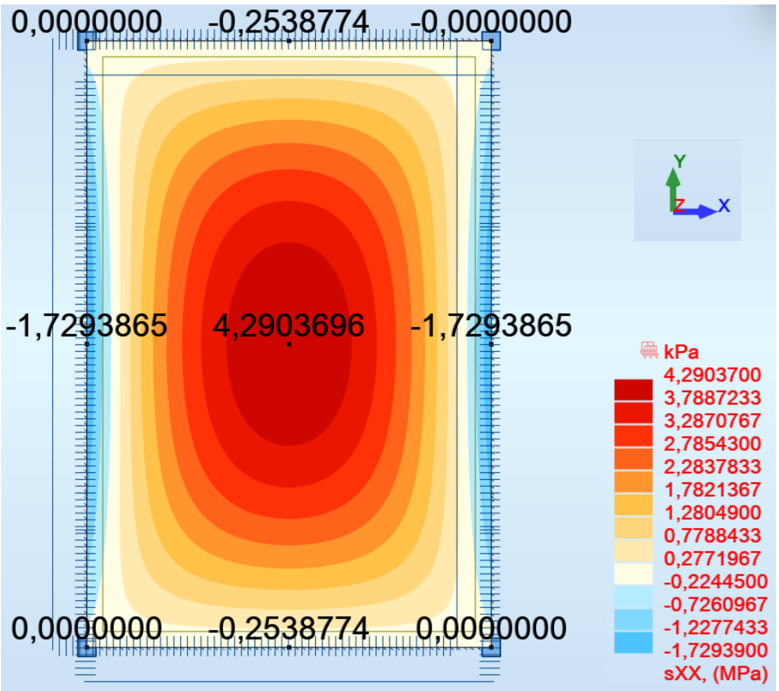


**Figure S4-2.** Map of stress $\sigma_x$ for the plate with reference parameters at $C$ = 1 kNm/(m·rad).

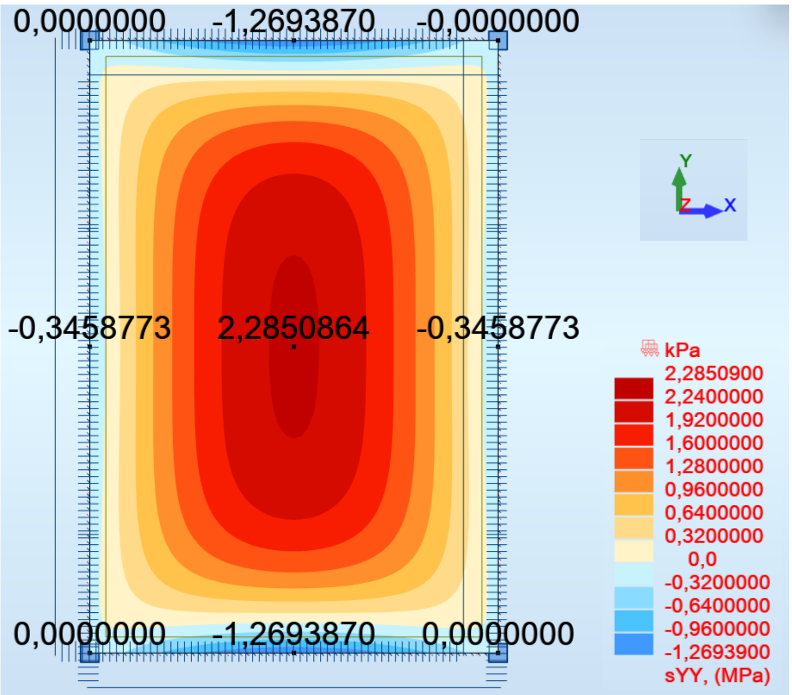


**Figure S4-3.** Map of stress $\sigma_y$ for the plate with reference parameters at $C = 1$ kNm/(m·rad).
