# Supplementary material for: The Influence of Elastic Support of Component Glass Panes on Deflection and Stress in Insulating Glass Units—Analytical Model
Source: Materials (Basel). 2024 Sep 23;17(18):4669. doi: 10.3390/ma17184669 (PMC11433723; doi:10.3390/ma17184669)
Supplement: Supplementary file 1 [file materials-17-04669-s001.zip › File S5.pdf]

File S5
Calculations for Symmetrically Loaded IGUs—Spreadsheet Printout.

Reference IGU parameters

Width (of IGU)	a =	60	cm
		0.6	m
Aspect ratio	ε =	1.5	
Glass pane thickness	d =	4	mm
		0.004	m
Gas cavity thickness	s =	16	mm
Young's modulus	E =	70	GPa
		70000000	kPa
Poisson's ratio	μ =	0.2	
Flexural rigidity	D =	0.38888889	kNm
Atmospheric pressure decrease	Δp =	5	kPa

"diff. w " means the percentage change in deflection at the pane center with respect to the case of simply support.

"diff. $\sigma_{x,cen}$" means percentage change in stress $\sigma_{x,cen}$ with respect to the case of simply support.

Other symbols are explained in the main text.

Data in green are presented in Figures 5–8 in the main text.

Table S5-1. The influence of rotational stiffness on static quantities in IGUs loaded with a 5 kPa atmospheric pressure decrease for various widths of the unit **(data related to Figure 5 in main text)**.

a [m]	C [kNm/(m*rad)]	R_c	α'_w	α'_v	$k_{x,cen}$	$k_{y,cen}$	$k_{x,ed} = G'$	$k_{y,ed} = F'$	q [kN/m^2]	r_{sym}	w [mm]	$\sigma_{x,cen}$ [MPa]	$\sigma_{y,cen}$ [MPa]	$\sigma_{x,ed}$ [MPa]	$\sigma_{y,ed}$ [MPa]	diff. w	diff. $\sigma_{x,cen}$
0.4	0.00000001	610865238	0.00772408	0.004911771	0.078419513	0.042579389	0	0	1.389975036	72.20%	0.7067539	6.540069946	3.551057261	0	0	0.00%	0.00%
0.4	0.1	61.0865238	0.007534977	0.004778947	0.076954346	0.041720874	-0.002486672	-0.001811215	1.417365561	71.65%	0.7030371	6.54434635	3.548023785	-0.2114714	-0.15402924	-0.53%	0.07%
0.4	0.2	30.5432619	0.007358466	0.004654986	0.075586956	0.040919014	-0.004810428	-0.003502695	1.443924612	71.12%	0.6994332	6.548511941	3.545038271	-0.4167537	-0.30345769	-1.04%	0.13%
0.4	0.5	12.2173048	0.006893031	0.00432822	0.071982424	0.03880199	-0.010951517	-0.007967846	1.51897819	69.62%	0.689249	6.56038395	3.536362556	-0.9981069	-0.7261791	-2.48%	0.31%
0.4	1	6.10865238	0.006280331	0.003898331	0.067240273	0.036008334	-0.019070937	-0.013860744	1.630546229	67.39%	0.6741089	6.578302394	3.522795226	-1.8657626	-1.35603506	-4.62%	0.58%
0.4	2	3.05432619	0.005437012	0.003307286	0.060720019	0.032146696	-0.03033114	-0.022018106	1.813909045	63.72%	0.6492176	6.608435542	3.498670969	-3.3010758	-2.39633048	-8.14%	1.05%
0.4	3.5	1.74532925	0.004672566	0.002772442	0.05481896	0.028623346	-0.040654017	-0.029499704	2.019747933	59.61%	0.621251	6.643228868	3.468716603	-4.926652	-3.57491794	-12.10%	1.58%
0.4	5	1.22173048	0.004201038	0.002443162	0.051185101	0.026435002	-0.047095586	-0.034191622	2.1717013	56.57%	0.6005804	6.669525073	3.44453569	-6.1366528	-4.45523944	-15.02%	1.98%
a [m]	C [kNm/(m*rad)]	R_c	α'_w	α'_v	$k_{x,cen}$	$k_{y,cen}$	$k_{x,ed} = G'$	$k_{y,ed} = F'$	q [kN/m^2]	r_{sym}	w [mm]	$\sigma_{x,cen}$ [MPa]	$\sigma_{y,cen}$ [MPa]	$\sigma_{x,ed}$ [MPa]	$\sigma_{y,ed}$ [MPa]	diff. w	diff. $\sigma_{x,cen}$
0.6	0.00000001	407243492	0.00772408	0.004911771	0.078419513	0.042579389	0	0	0.356955514	92.86%	0.9188409	3.778957483	2.051857934	0	0	0.00%	0.00%
0.6	0.1	40.7243492	0.007445228	0.004715915	0.076259051	0.041313221	-0.003667884	-0.002671159	0.370631977	92.59%	0.9196029	3.815645783	2.067120113	-0.1835237	-0.13365228	0.08%	0.97%
0.6	0.2	20.3621746	0.007193328	0.004539031	0.074307865	0.04016834	-0.006986949	-0.005086059	0.383916823	92.32%	0.9203361	3.851285345	2.081875698	-0.362125	-0.26360419	0.16%	1.91%
0.6	0.5	8.14486984	0.006565156	0.004098131	0.069444304	0.03730808	-0.015290971	-0.011118763	0.421584387	91.57%	0.9223782	3.952345659	2.123348068	-0.8702687	-0.63281207	0.38%	4.59%
0.6	1	4.07243492	0.005809797	0.003568446	0.063601139	0.03385644	-0.025339679	-0.018403486	0.47792272	90.44%	0.9253332	4.103517958	2.184402831	-1.6349051	-1.18738496	0.71%	8.59%
0.6	2	2.03621746	0.004883634	0.002920006	0.056447186	0.029598842	-0.037790434	-0.027421823	0.571418981	88.57%	0.9299878	4.354424133	2.283300928	-2.9152131	-2.11536229	1.21%	15.23%
0.6	3.5	1.16355283	0.00413945	0.002400198	0.05071089	0.026148127	-0.047941969	-0.034810983	0.677728711	86.45%	0.9349277	4.639710518	2.392380371	-4.3863726	-3.18497437	1.75%	22.78%
0.6	5	0.81448698	0.003719041	0.002107252	0.047476796	0.024182183	-0.053754977	-0.03909573	0.757135168	84.86%	0.9383911	4.852757514	2.471739511	-5.4944708	-3.99610149	2.13%	28.42%
a [m]	C [kNm/(m*rad)]	R_c	α'_w	α'_v	$k_{x,cen}$	$k_{y,cen}$	$k_{x,ed} = G'$	$k_{y,ed} = F'$	q [kN/m^2]	r_{sym}	w [mm]	$\sigma_{x,cen}$ [MPa]	$\sigma_{y,cen}$ [MPa]	$\sigma_{x,ed}$ [MPa]	$\sigma_{y,ed}$ [MPa]	diff. w	diff. $\sigma_{x,cen}$
0.8	0.00000001	305432619	0.00772408	0.004911771	0.078419513	0.042579389	0	0	0.119027588	97.62%	0.9683421	2.240180516	1.216349268	0	0	0.00%	0.00%
0.8	0.1	30.5432619	0.007358466	0.004654986	0.075586956	0.040919014	-0.004810428	-0.003502695	0.125420638	97.49%	0.9720547	2.275239418	1.231701322	-0.1447985	-0.10543447	0.38%	1.57%
0.8	0.2	15.271631	0.007038495	0.004430329	0.073108783	0.039464067	-0.009029959	-0.006571467	0.131604848	97.37%	0.9756322	2.309152867	1.246479007	-0.2852127	-0.20756088	0.75%	3.08%
0.8	0.5	6.10865238	0.006280331	0.003898331	0.067240273	0.036008334	-0.019070937	-0.013860744	0.149002939	97.02%	0.9856252	2.404559589	1.287683438	-0.6819901	-0.49566999	1.78%	7.34%
0.8	1	3.05432619	0.005437012	0.003307286	0.060720019	0.032146696	-0.03033114	-0.022018106	0.174655444	96.51%	1.000177	2.545219663	1.347502914	-1.2713997	-0.92293969	3.29%	13.62%
0.8	2	1.5271631	0.004492399	0.00264656	0.053429861	0.027788774	-0.043107652	-0.031283225	0.216281547	95.67%	1.0233688	2.773414302	1.442447774	-2.2376135	-1.62383625	5.68%	23.80%
0.8	3.5	0.87266463	0.003795878	0.002160745	0.048067467	0.024542565	-0.052687699	-0.038304015	0.262238061	94.76%	1.0484371	3.025228646	1.544638728	-3.3160128	-2.41074492	8.27%	35.04%
0.8	5	0.61086524	0.003424422	0.001902366	0.045213845	0.022795296	-0.057868998	-0.042178631	0.295650846	94.09%	1.0663526	3.208202804	1.617467661	-4.1061644	-2.99283552	10.12%	43.21%
a [m]	C [kNm/(m*rad)]	R_c	α'_w	α'_v	$k_{x,cen}$	$k_{y,cen}$	$k_{x,ed} = G'$	$k_{y,ed} = F'$	q [kN/m^2]	r_{sym}	w [mm]	$\sigma_{x,cen}$ [MPa]	$\sigma_{y,cen}$ [MPa]	$\sigma_{x,ed}$ [MPa]	$\sigma_{y,ed}$ [MPa]	diff. w	diff. $\sigma_{x,cen}$
1.0	0.00000001	244346095	0.00772408	0.004911771	0.078419513	0.042579389	0	0	0.049484511	99.01%	0.9828574	1.455206736	0.790132596	0	0	0.00%	0.00%
1.0	0.1	24.4346095	0.007274546	0.004596057	0.074936917	0.040537592	-0.005916193	-0.004307225	0.052845947	98.94%	0.9885349	1.485042139	0.803342805	-0.1172426	-0.08535728	0.58%	2.05%
1.0	0.2	12.2173048	0.006893031	0.00432822	0.071982424	0.03880199	-0.010951517	-0.007967846	0.056077576	98.88%	0.9939714	1.513724951	0.815970571	-0.2303005	-0.16755657	1.13%	4.02%
1.0	0.5	4.88692191	0.006030584	0.003723206	0.06530839	0.034866949	-0.022394193	-0.016269607	0.065065415	98.70%	1.0089835	1.593494049	0.850737188	-0.5464078	-0.39697078	2.66%	9.50%
1.0	1	2.44346095	0.005134329	0.003095388	0.058382231	0.030754699	-0.034403074	-0.024966985	0.078045713	98.44%	1.0304031	1.708681076	0.900102157	-1.0068796	-0.73071232	4.84%	17.42%
1.0	2	1.22173048	0.004201038	0.002443162	0.051185101	0.026435002	-0.047095586	-0.034191622	0.098449749	98.03%	1.06352	1.889685136	0.975944737	-1.7387057	-1.26230873	8.21%	29.86%
1.0	3.5	0.6981317	0.00355589	0.001993745	0.046223254	0.023415197	-0.056028869	-0.040792786	0.12008189	97.60%	1.0979947	2.0814659	1.054402939	-2.5230197	-1.83692809	11.71%	43.04%
1.0	5	0.48869219	0.003225577	0.001764318	0.043688364	0.021854251	-0.060665761	-0.044312989	0.135253533	97.29%	1.121839	2.215877076	1.108449269	-3.076972	-2.24755812	14.14%	52.27%
a [m]	C [kNm/(m*rad)]	R_c	α'_w	α'_v	$k_{x,cen}$	$k_{y,cen}$	$k_{x,ed} = G'$	$k_{y,ed} = F'$	q [kN/m^2]	r_{sym}	w [mm]	$\sigma_{x,cen}$ [MPa]	$\sigma_{y,cen}$ [MPa]	$\sigma_{x,ed}$ [MPa]	$\sigma_{y,ed}$ [MPa]	diff. w	diff. $\sigma_{x,cen}$
1.2	0.00000001	203621746	0.00772408	0.004911771	0.078419513	0.042579389	0	0	0.023993374	99.52%	0.9881833	1.016036287	0.551676521	0	0	0.00%	0.00%
1.2	0.1	20.3621746	0.007193328	0.004539031	0.074307865	0.04016834	-0.006986949	-0.005086059	0.025952927	99.48%	0.9954413	1.041393565	0.562942436	-0.0979192	-0.07127898	0.73%	2.50%
1.2	0.2	10.1810873	0.006756107	0.004232121	0.070922363	0.038178397	-0.012762269	-0.009283068	0.027823988	99.44%	1.0023406	1.065605214	0.573628653	-0.1917525	-0.13947767	1.43%	4.88%
1.2	0.5	4.07243492	0.005809797	0.003568446	0.063601139	0.03385644	-0.025339679	-0.018403486	0.03296293	99.34%	1.0211422	1.132099139	0.602644021	-0.4510458	-0.32758172	3.34%	11.42%
1.2	1	2.03621746	0.004883634	0.002920006	0.056447186	0.029598842	-0.037790434	-0.027421823	0.040221013	99.20%	1.047359	1.225996029	0.642867526	-0.8207836	-0.5955841	5.99%	20.66%
1.2	2	1.01810873	0.003975572	0.002285933	0.049449578	0.025383443	-0.050200357	-0.036468412	0.05125725	98.97%	1.0865616	1.368710656	0.702586161	-1.3894914	-1.00940609	9.96%	34.71%
1.2	3.5	0.58177642	0.003378713	0.001870615	0.04486304	0.022579351	-0.058510454	-0.04266488	0.062487976	98.75%	1.1257635	1.513836288	0.761906503	-1.9743479	-1.43966269	13.92%	48.99%
1.2	5	0.40724349	0.003082283	0.00166497	0.042590024	0.021173325	-0.062691214	-0.045886114	0.070092277	98.60%	1.1519724	1.612025163	0.801406729	-2.3728518	-1.73678161	16.57%	58.66%

Table S5-2. The influence of rotational stiffness on static quantities in IGUs loaded with a 5 kPa atmospheric pressure decrease for various aspect ratios of the unit **(data related to Figure 6 in main text)**.

ε	C [kNm/(m*rad)]	R_c	α'_w	α'_v	$k_{x,cen}$	$k_{y,cen}$	$k_{x,ed} = G'$	$k_{y,ed} = F'$	q [kN/m^2]	r_{sym}	w [mm]	$\sigma_{x,cen}$ [MPa]	$\sigma_{y,cen}$ [MPa]	$\sigma_{x,ed}$ [MPa]	$\sigma_{y,ed}$ [MPa]	diff. w	diff. $\sigma_{x,cen}$
1.0	0.00000001	407243492	0.00406241	0.001702503	0.044263846	0.044215019	0	0	0.642417872	87.15%	0.8697227	3.838844543	3.834609963	0	0	0.00%	0.00%
1.0	0.1	40.7243492	0.003944625	0.001646959	0.043296025	0.043247198	-0.001993806	-0.001993806	0.661106299	86.78%	0.8690735	3.864142084	3.859784318	-0.1779459	-0.17794592	-0.07%	0.66%
1.0	0.2	20.3621746	0.003836424	0.001595939	0.042406887	0.04235806	-0.003828841	-0.003828841	0.67925799	86.41%	0.868442	3.888704282	3.884226867	-0.3511041	-0.35110406	-0.15%	1.30%
1.0	0.5	8.14486984	0.003558883	0.001465093	0.040125883	0.040077056	-0.008553314	-0.008553314	0.730716582	85.39%	0.8666467	3.958287504	3.953470893	-0.8437565	-0.84375654	-0.35%	3.11%
1.0	1	4.07243492	0.003209807	0.001300583	0.037256134	0.037207308	-0.014540556	-0.014540556	0.807660323	83.85%	0.8639471	4.062190711	4.056866915	-1.5854171	-1.58541711	-0.66%	5.82%
1.0	2	2.03621746	0.002757237	0.001087444	0.033533533	0.033484707	-0.022411338	-0.022411338	0.935304569	81.29%	0.8594222	4.234149057	4.227983878	-2.8297926	-2.8297926	-1.18%	10.30%
1.0	3.5	1.16355283	0.002372438	0.000906432	0.030365498	0.030316671	-0.029255956	-0.029255956	1.080395093	78.39%	0.8541949	4.428909248	4.421787686	-4.2670789	-4.26707886	-1.79%	15.37%
1.0	5	0.81448698	0.002146139	0.000800125	0.028500386	0.028451559	-0.033384547	-0.033384547	1.188760795	76.22%	0.850221	4.5738191	4.565983232	-5.3576425	-5.3576425	-2.24%	19.15%
ε	C [kNm/(m*rad)]	R_c	α'_w	α'_v	$k_{x,cen}$	$k_{y,cen}$	$k_{x,ed} = G'$	$k_{y,ed} = F'$	q [kN/m^2]	r_{sym}	w [mm]	$\sigma_{x,cen}$ [MPa]	$\sigma_{y,cen}$ [MPa]	$\sigma_{x,ed}$ [MPa]	$\sigma_{y,ed}$ [MPa]	diff. w	diff. $\sigma_{x,cen}$
1.2	0.00000001	407243492	0.005650587	0.002848026	0.059305886	0.04485606	0	0	0.478939343	90.42%	0.90189	3.834529478	2.900249803	0	0	0.00%	0.00%
1.2	0.1	40.7243492	0.00547072	0.00274641	0.057888214	0.04374899	-0.002724864	-0.002351929	0.494831294	90.10%	0.9021554	3.867061475	2.92252985	-0.182027	-0.15711409	0.03%	0.85%
1.2	0.2	20.3621746	0.005306727	0.002653776	0.05659586	0.042739072	-0.005214832	-0.00449939	0.510267421	89.79%	0.9024103	3.89866818	2.944128095	-0.3592295	-0.30994546	0.06%	1.67%
1.2	0.5	8.14486984	0.004891376	0.002419219	0.053323914	0.040178712	-0.011548354	-0.009953786	0.554030828	88.92%	0.9031178	3.98831747	3.005133114	-0.8637495	-0.74448506	0.14%	4.01%
1.2	1	4.07243492	0.004379529	0.002130326	0.0492947	0.037017278	-0.019420483	-0.016716104	0.619477245	87.61%	0.9041329	4.122487534	3.095733815	-1.6241239	-1.39795825	0.25%	7.51%
1.2	2	2.03621746	0.003732766	0.001765641	0.044209618	0.033008637	-0.029518894	-0.025362429	0.728071657	85.44%	0.9057	4.345348912	3.244408204	-2.9014024	-2.49286484	0.42%	13.32%
1.2	3.5	1.16355283	0.003197156	0.001464109	0.040005941	0.029671271	-0.038077058	-0.032672529	0.851541884	82.97%	0.9072968	4.599009134	3.410954574	-4.3772683	-3.75597367	0.60%	19.94%
1.2	5	0.81448698	0.002888057	0.001290405	0.037584275	0.02773451	-0.043136699	-0.036997746	0.943779302	81.12%	0.9083552	4.788620247	3.533659581	-5.4960556	-4.71389038	0.72%	24.88%
ε	C [kNm/(m*rad)]	R_c	α'_w	α'_v	$k_{x,cen}$	$k_{y,cen}$	$k_{x,ed} = G'$	$k_{y,ed} = F'$	q [kN/m^2]	r_{sym}	w [mm]	$\sigma_{x,cen}$ [MPa]	$\sigma_{y,cen}$ [MPa]	$\sigma_{x,ed}$ [MPa]	$\sigma_{y,ed}$ [MPa]	diff. w	diff. $\sigma_{x,cen}$
1.5	0.00000001	407243492	0.00772408	0.004911771	0.078419513	0.042579389	0	0	0.356955514	92.86%	0.9188409	3.778957483	2.051857934	0	0	0.00%	0.00%
1.5	0.1	40.7243492	0.007445228	0.004715915	0.076259051	0.041313221	-0.003667884	-0.002671159	0.370631977	92.59%	0.9196029	3.815645783	2.067120113	-0.1835237	-0.13365228	0.08%	0.97%
1.5	0.2	20.3621746	0.007193328	0.004539031	0.074307865	0.04016834	-0.006986949	-0.005086059	0.383916823	92.32%	0.9203361	3.851285345	2.081875698	-0.362125	-0.26360419	0.16%	1.91%
1.5	0.5	8.14486984	0.006565156	0.004098131	0.069444304	0.03730808	-0.015290971	-0.011118763	0.421584387	91.57%	0.9223782	3.952345659	2.123348068	-0.8702687	-0.63281207	0.38%	4.59%
1.5	1	4.07243492	0.005809797	0.003568446	0.063601139	0.03385644	-0.025339679	-0.018403486	0.47792272	90.44%	0.9253332	4.103517958	2.184402831	-1.6349051	-1.18738496	0.71%	8.59%
1.5	2	2.03621746	0.004883634	0.002920006	0.056447186	0.029598842	-0.037790434	-0.027421823	0.571418981	88.57%	0.9299878	4.354424133	2.283300928	-2.9152131	-2.11536229	1.21%	15.23%
1.5	3.5	1.16355283	0.00413945	0.002400198	0.05071089	0.026148127	-0.047941969	-0.034810983	0.677728711	86.45%	0.9349277	4.639710518	2.392380371	-4.3863726	-3.18497437	1.75%	22.78%
1.5	5	0.81448698	0.003719041	0.002107252	0.047476796	0.024182183	-0.053754977	-0.03909573	0.757135168	84.86%	0.9383911	4.852757514	2.471739511	-5.4944708	-3.99610149	2.13%	28.42%
ε	C [kNm/(m*rad)]	R_c	α'_w	α'_v	$k_{x,cen}$	$k_{y,cen}$	$k_{x,ed} = G'$	$k_{y,ed} = F'$	q [kN/m^2]	r_{sym}	w [mm]	$\sigma_{x,cen}$ [MPa]	$\sigma_{y,cen}$ [MPa]	$\sigma_{x,ed}$ [MPa]	$\sigma_{y,ed}$ [MPa]	diff. w	diff. $\sigma_{x,cen}$
2.0	0.00000001	407243492	0.01012872	0.008808206	0.100002869	0.03671657	0	0	0.270583791	94.59%	0.913347	3.652985989	1.341212658	0	0	0.00%	0.00%
2.0	0.1	40.7243492	0.009699354	0.008408809	0.096660113	0.035307718	-0.004742796	-0.002877449	0.282675284	94.35%	0.9137137	3.688662345	1.347383611	-0.1809906	-0.10980678	0.04%	0.98%
2.0	0.2	20.3621746	0.009315999	0.008052252	0.093675625	0.034049542	-0.008976684	-0.005452042	0.294420972	94.11%	0.9140661	3.72330924	1.353361408	-0.3567947	-0.21670141	0.08%	1.93%
2.0	0.5	8.14486984	0.008378243	0.007180234	0.086375305	0.03097055	-0.019329562	-0.011777698	0.327726056	93.45%	0.915047	3.821504152	1.370230599	-0.8551981	-0.52108089	0.19%	4.61%
2.0	1	4.07243492	0.007284073	0.006163161	0.077857745	0.027375498	-0.031397187	-0.019230083	0.37754089	92.45%	0.9164689	3.968255113	1.395274958	-1.6002524	-0.98011925	0.34%	8.63%
2.0	2	2.03621746	0.005990499	0.004961442	0.067788222	0.023121821	-0.045628361	-0.028214849	0.460200353	90.80%	0.9187332	4.211482106	1.436490466	-2.8347554	-1.75290527	0.59%	15.29%
2.0	3.5	1.16355283	0.004988341	0.004031114	0.059986498	0.019825056	-0.056593394	-0.035425892	0.554142496	88.92%	0.9212066	4.487544162	1.483097297	-4.2337086	-2.65018391	0.86%	22.85%
2.0	5	0.81448698	0.004436744	0.003519334	0.05569133	0.018011468	-0.062581858	-0.03956913	0.624257354	87.51%	0.9230124	4.693372506	1.517911833	-5.27407	-3.33467825	1.06%	28.48%

Table S5-3. The influence of rotational stiffness on static quantities in IGUs loaded with a 5 kPa atmospheric pressure decrease for various thicknesses of glass panes **(data related to Figure 7 in main text)**.

d [m]	D [kNm]	C [kNm/(m*rad)]	R_c	α'_w	α'_v	$k_{x,cen}$	$k_{y,cen}$	$k_{x,ed} = G'$	$k_{y,ed} = F'$	q [kN/m^2]	r_{sym}	w [mm]	$\sigma_{x,cen}$ [MPa]	$\sigma_{y,cen}$ [MPa]	$\sigma_{x,ed}$ [MPa]	$\sigma_{y,ed}$ [MPa]	diff. w	diff. $\sigma_{x,cen}$
0.004	0.38888889	0.00000001	407243492	0.00772408	0.004911771	0.078419513	0.042579389	0	0	0.356955514	92.86%	0.9188409	3.778957483	2.051857934	0	0	0.00%	0.00%
0.004	0.38888889	0.1	40.7243492	0.007445228	0.004715915	0.076259051	0.041313221	-0.003667884	-0.002671159	0.370631977	92.59%	0.9196029	3.815645783	2.067120113	-0.1835237	-0.13365228	0.08%	0.97%
0.004	0.38888889	0.2	20.3621746	0.007193328	0.004539031	0.074307865	0.04016834	-0.006986949	-0.005086059	0.383916823	92.32%	0.9203361	3.851285345	2.081875698	-0.362125	-0.26360419	0.16%	1.91%
0.004	0.38888889	0.5	8.14486984	0.006565156	0.004098131	0.069444304	0.03730808	-0.015290971	-0.011118763	0.421584387	91.57%	0.9223782	3.952345659	2.123348068	-0.8702687	-0.63281207	0.38%	4.59%
0.004	0.38888889	1	4.07243492	0.005809797	0.003568446	0.063601139	0.03385644	-0.025339679	-0.018403486	0.47792272	90.44%	0.9253332	4.103517958	2.184402831	-1.6349051	-1.18738496	0.71%	8.59%
0.004	0.38888889	2	2.03621746	0.004883634	0.002920006	0.056447186	0.029598842	-0.037790434	-0.027421823	0.571418981	88.57%	0.9299878	4.354424133	2.283300928	-2.9152131	-2.11536229	1.21%	15.23%
0.004	0.38888889	3.5	1.16355283	0.00413945	0.002400198	0.05071089	0.026148127	-0.047941969	-0.034810983	0.677728711	86.45%	0.9349277	4.639710518	2.392380371	-4.3863726	-3.18497437	1.75%	22.78%
0.004	0.38888889	5	0.81448698	0.003719041	0.002107252	0.047476796	0.024182183	-0.053754977	-0.03909573	0.757135168	84.86%	0.9383911	4.852757514	2.471739511	-5.4944708	-3.99610149	2.13%	28.42%
d [m]	D [kNm]	C [kNm/(m*rad)]	R_c	α'_w	α'_v	$k_{x,cen}$	$k_{y,cen}$	$k_{x,ed} = G'$	$k_{y,ed} = F'$	q [kN/m^2]	r_{sym}	w [mm]	$\sigma_{x,cen}$ [MPa]	$\sigma_{y,cen}$ [MPa]	$\sigma_{x,ed}$ [MPa]	$\sigma_{y,ed}$ [MPa]	diff. w	diff. $\sigma_{x,cen}$
0.006	1.3125	0.00000001	1374446786	0.00772408	0.004911771	0.078419513	0.042579389	0	0	1.024379383	79.51%	0.7812918	4.819879957	2.617046891	0	0	0.00%	0.00%
0.006	1.3125	0.1	137.444679	0.007638394	0.004851584	0.077755595	0.042190451	-0.001126394	-0.000820577	1.034372992	79.31%	0.7801622	4.825697239	2.618439759	-0.0699067	-0.05092698	-0.14%	0.12%
0.006	1.3125	0.2	68.7223393	0.007555342	0.00479325	0.077112127	0.041813359	-0.002218728	-0.00161611	1.044247046	79.12%	0.779046	4.831446672	2.619808614	-0.139014	-0.10125708	-0.29%	0.24%
0.006	1.3125	0.5	27.4889357	0.007320826	0.004628554	0.075295392	0.040747952	-0.005306311	-0.003863519	1.073173161	78.54%	0.7757746	4.848299609	2.623776527	-0.3416755	-0.24877346	-0.71%	0.59%
0.006	1.3125	1	13.7444679	0.006972739	0.004384169	0.072599601	0.039164832	-0.009898316	-0.007202581	1.119183461	77.62%	0.770567	4.875136384	2.629957943	-0.6646819	-0.48366058	-1.37%	1.15%
0.006	1.3125	2	6.87223393	0.006402165	0.003983787	0.068182951	0.036564545	-0.017452803	-0.012687242	1.203762485	75.92%	0.7609802	4.92456469	2.640901617	-1.2605417	-0.91634553	-2.60%	2.17%
0.006	1.3125	3.5	3.92699082	0.005764165	0.003536468	0.063248361	0.033647401	-0.025949406	-0.018845101	1.314831412	73.70%	0.7483628	4.989655888	2.654439587	-2.0471457	-1.48668784	-4.21%	3.52%
0.006	1.3125	5	2.74889357	0.005295075	0.003207902	0.059623577	0.031494402	-0.032238295	-0.023399118	1.41047915	71.79%	0.7374702	5.045868727	2.665331878	-2.7282866	-1.98023812	-5.61%	4.69%
d [m]	D [kNm]	C [kNm/(m*rad)]	R_c	α'_w	α'_v	$k_{x,cen}$	$k_{y,cen}$	$k_{x,ed} = G'$	$k_{y,ed} = F'$	q [kN/m^2]	r_{sym}	w [mm]	$\sigma_{x,cen}$ [MPa]	$\sigma_{y,cen}$ [MPa]	$\sigma_{x,ed}$ [MPa]	$\sigma_{y,ed}$ [MPa]	diff. w	diff. $\sigma_{x,cen}$
0.008	3.11111111	0.00000001	3257947937	0.00772408	0.004911771	0.078419513	0.042579389	0	0	1.885384113	62.29%	0.606647	4.989968023	2.709399491	0	0	0.00%	0.00%
0.008	3.11111111	0.1	325.794794	0.007687601	0.004886148	0.078136867	0.042413825	-0.000479454	-0.000349311	1.891457163	62.17%	0.6057269	4.987998087	2.707557743	-0.0306068	-0.02229888	-0.15%	-0.04%
0.008	3.11111111	0.2	162.897397	0.007651605	0.004860864	0.07785796	0.042250427	-0.000952681	-0.000694043	1.897488623	62.05%	0.6048131	4.986042497	2.705727542	-0.0610099	-0.04444668	-0.30%	-0.08%
0.008	3.11111111	0.5	65.1589587	0.007546413	0.004786979	0.077042949	0.04177281	-0.002336202	-0.001701651	1.915337729	61.69%	0.6021094	4.980260232	2.700305094	-0.1510183	-0.10999924	-0.75%	-0.19%
0.008	3.11111111	1	32.5794794	0.007379885	0.004670027	0.075752868	0.041016342	-0.004528311	-0.003297396	1.944293386	61.11%	0.5977242	4.970895783	2.691488332	-0.2971473	-0.21637482	-1.47%	-0.38%
0.008	3.11111111	2	16.2897397	0.007076291	0.004456862	0.073401467	0.039636024	-0.008531037	-0.006208791	1.99940541	60.01%	0.5893808	4.953126044	2.674636237	-0.5756738	-0.41896882	-2.85%	-0.74%
0.008	3.11111111	3.5	9.30842268	0.006682562	0.004180511	0.070353049	0.03784329	-0.013735707	-0.009989865	2.075725297	58.49%	0.577833	4.928634128	2.65113925	-0.9622649	-0.6998473	-4.75%	-1.23%
0.008	3.11111111	5	6.51589587	0.006347974	0.003945775	0.067763631	0.03631719	-0.018172308	-0.013209093	2.145328246	57.09%	0.5673073	4.906414059	2.629539873	-1.3157628	-0.95640208	-6.48%	-1.67%
d [m]	D [kNm]	C [kNm/(m*rad)]	R_c	α'_w	α'_v	$k_{x,cen}$	$k_{y,cen}$	$k_{x,ed} = G'$	$k_{y,ed} = F'$	q [kN/m^2]	r_{sym}	w [mm]	$\sigma_{x,cen}$ [MPa]	$\sigma_{y,cen}$ [MPa]	$\sigma_{x,ed}$ [MPa]	$\sigma_{y,ed}$ [MPa]	diff. w	diff. $\sigma_{x,cen}$
0.01	6.07638889	0.00000001	6363179565	0.00772408	0.004911771	0.078419513	0.042579389	0	0	2.6984557	46.03%	0.4445509	4.570810181	2.481809647	0	0	0.00%	0.00%
0.01	6.07638889	0.1	636.317956	0.007705342	0.00489861	0.078274326	0.042494347	-0.000246266	-0.000179425	2.701746467	45.97%	0.4440133	4.567911505	2.479873355	-0.0143715	-0.01047085	-0.12%	-0.06%
0.01	6.07638889	0.2	318.158978	0.007686732	0.004885537	0.078130129	0.042409878	-0.000490884	-0.000357638	2.70502287	45.90%	0.443478	4.56502579	2.477945318	-0.0286816	-0.02089627	-0.24%	-0.13%
0.01	6.07638889	0.5	127.263591	0.007631654	0.00484685	0.077703378	0.042159855	-0.001215013	-0.000885126	2.714766836	45.70%	0.4418864	4.556445535	2.472210187	-0.0712471	-0.05190287	-0.60%	-0.31%
0.01	6.07638889	1	63.6317956	0.00754229	0.004784083	0.077011007	0.041754088	-0.002390444	-0.001741148	2.730728154	45.39%	0.4392797	4.542396322	2.462811756	-0.1409973	-0.10269941	-1.19%	-0.62%
0.01	6.07638889	2	31.8158978	0.007372153	0.004664598	0.07569298	0.040981211	-0.00463014	-0.0033715	2.761645657	44.77%	0.4342319	4.515203289	2.444592612	-0.276195	-0.20111517	-2.32%	-1.22%
0.01	6.07638889	3.5	18.180513	0.007136453	0.004499099	0.073867379	0.039909687	-0.00773715	-0.0056316	2.805663324	43.89%	0.4270487	4.476535126	2.418619958	-0.4688893	-0.34128807	-3.94%	-2.06%
0.01	6.07638889	5	12.7263591	0.006921415	0.004348143	0.072202198	0.038931214	-0.010576392	-0.007695301	2.847075342	43.06%	0.4202941	4.4402061	2.394146149	-0.6504146	-0.47323661	-5.46%	-2.86%

Table S5-4. The influence of rotational stiffness on static quantities in IGUs loaded with a 5 kPa atmospheric pressure decrease for various thicknesses of gas cavity **(data related to Figure 8 in main text)**.

s [m]	C [kNm/(m*rad)]	R_c	α'_w	α'_v	$k_{x,cen}$	$k_{y,cen}$	$k_{x,ed} = G'$	$k_{y,ed} = F'$	q [kN/m^2]	r_{sym}	w [mm]	$\sigma_{x,cen}$ [MPa]	$\sigma_{y,cen}$ [MPa]	$\sigma_{x,ed}$ [MPa]	$\sigma_{y,ed}$ [MPa]	diff. w	diff. $\sigma_{x,cen}$
0.012	0.00000001	407243492	0.00772408	0.004911771	0.078419513	0.042579389	0	0	0.272809223	94.54%	0.7022395	2.888131476	1.568166753	0	0	0.00%	0.00%
0.012	0.1	40.7243492	0.007445228	0.004715915	0.076259051	0.041313221	-0.003667884	-0.002671159	0.283466952	94.33%	0.7033312	2.9182843	1.580975938	-0.1403627	-0.10222001	0.16%	1.04%
0.012	0.2	20.3621746	0.007193328	0.004539031	0.074307865	0.04016834	-0.006986949	-0.005086059	0.293834208	94.12%	0.7043876	2.947616021	1.593382367	-0.2771556	-0.20175185	0.31%	2.06%
0.012	0.5	8.14486984	0.006565156	0.004098131	0.069444304	0.03730808	-0.015290971	-0.011118763	0.323308281	93.53%	0.7073613	3.031009019	1.628371529	-0.6673992	-0.48529639	0.73%	4.95%
0.012	1	4.07243492	0.005809797	0.003568446	0.063601139	0.03385644	-0.025339679	-0.018403486	0.367610642	92.65%	0.7117517	3.156361496	1.680208314	-1.2575433	-0.91331784	1.35%	9.29%
0.012	2	2.03621746	0.004883634	0.002920006	0.056447186	0.029598842	-0.037790434	-0.027421823	0.441715423	91.17%	0.7188945	3.366035019	1.765025787	-2.2535034	-1.6352067	2.37%	16.55%
0.012	3.5	1.16355283	0.00413945	0.002400198	0.05071089	0.026148127	-0.047941969	-0.034810983	0.526868363	89.46%	0.7268157	3.606925079	1.859843783	-3.4099794	-2.47600877	3.50%	24.89%
0.012	5	0.81448698	0.003719041	0.002107252	0.047476796	0.024182183	-0.053754977	-0.03909573	0.591098163	88.18%	0.7326054	3.788565333	1.929695971	-4.2895532	-3.11977088	4.32%	31.18%
s [m]	C [kNm/(m*rad)]	R_c	α'_w	α'_v	$k_{x,cen}$	$k_{y,cen}$	$k_{x,ed} = G'$	$k_{y,ed} = F'$	q [kN/m^2]	r_{sym}	w [mm]	$\sigma_{x,cen}$ [MPa]	$\sigma_{y,cen}$ [MPa]	$\sigma_{x,ed}$ [MPa]	$\sigma_{y,ed}$ [MPa]	diff. w	diff. $\sigma_{x,cen}$
0.014	0.00000001	407243492	0.00772408	0.004911771	0.078419513	0.042579389	0	0	0.315277538	93.69%	0.8115574	3.337727992	1.812283862	0	0	0.00%	0.00%
0.014	0.1	40.7243492	0.007445228	0.004715915	0.076259051	0.041313221	-0.003667884	-0.002671159	0.327474498	93.45%	0.8125216	3.371340752	1.826418559	-0.1621537	-0.11808942	0.12%	1.01%
0.014	0.2	20.3621746	0.007193328	0.004539031	0.074307865	0.04016834	-0.006986949	-0.005086059	0.339330525	93.21%	0.8134526	3.404015143	1.840096427	-0.3200695	-0.23299044	0.23%	1.99%
0.014	0.5	8.14486984	0.006565156	0.004098131	0.069444304	0.03730808	-0.015290971	-0.011118763	0.372991451	92.54%	0.8160624	3.496787806	1.878605333	-0.7699592	-0.55987247	0.56%	4.77%
0.014	1	4.07243492	0.005809797	0.003568446	0.063601139	0.03385644	-0.025339679	-0.018403486	0.423460374	91.53%	0.8198855	3.635895885	1.935476181	-1.4485972	-1.05207486	1.03%	8.93%
0.014	2	2.03621746	0.004883634	0.002920006	0.056447186	0.029598842	-0.037790434	-0.027421823	0.507542549	89.85%	0.8260285	3.867662084	2.028060693	-2.5893342	-1.87889518	1.78%	15.88%
0.014	3.5	1.16355283	0.00413945	0.002400198	0.05071089	0.026148127	-0.047941969	-0.034810983	0.603644354	87.93%	0.8327283	4.132531217	2.130862805	-3.9068863	-2.83681621	2.61%	23.81%
0.014	5	0.81448698	0.003719041	0.002107252	0.047476796	0.024182183	-0.053754977	-0.03909573	0.675771782	86.48%	0.8375496	4.33126967	2.206121024	-4.904023	-3.56667176	3.20%	29.77%
s [m]	C [kNm/(m*rad)]	R_c	α'_w	α'_v	$k_{x,cen}$	$k_{y,cen}$	$k_{x,ed} = G'$	$k_{y,ed} = F'$	q [kN/m^2]	r_{sym}	w [mm]	$\sigma_{x,cen}$ [MPa]	$\sigma_{y,cen}$ [MPa]	$\sigma_{x,ed}$ [MPa]	$\sigma_{y,ed}$ [MPa]	diff. w	diff. $\sigma_{x,cen}$
0.016	0.00000001	407243492	0.00772408	0.004911771	0.078419513	0.042579389	0	0	0.356955514	92.86%	0.9188409	3.778957483	2.051857934	0	0	0.00%	0.00%
0.016	0.1	40.7243492	0.007445228	0.004715915	0.076259051	0.041313221	-0.003667884	-0.002671159	0.370631977	92.59%	0.9196029	3.815645783	2.067120113	-0.1835237	-0.13365228	0.08%	0.97%
0.016	0.2	20.3621746	0.007193328	0.004539031	0.074307865	0.04016834	-0.006986949	-0.005086059	0.383916823	92.32%	0.9203361	3.851285345	2.081875698	-0.362125	-0.26360419	0.16%	1.91%
0.016	0.5	8.14486984	0.006565156	0.004098131	0.069444304	0.03730808	-0.015290971	-0.011118763	0.421584387	91.57%	0.9223782	3.952345659	2.123348068	-0.8702687	-0.63281207	0.38%	4.59%
0.016	1	4.07243492	0.005809797	0.003568446	0.063601139	0.03385644	-0.025339679	-0.018403486	0.47792272	90.44%	0.9253332	4.103517958	2.184402831	-1.6349051	-1.18738496	0.71%	8.59%
0.016	2	2.03621746	0.004883634	0.002920006	0.056447186	0.029598842	-0.037790434	-0.027421823	0.571418981	88.57%	0.9299878	4.354424133	2.283300928	-2.9152131	-2.11536229	1.21%	15.23%
0.016	3.5	1.16355283	0.00413945	0.002400198	0.05071089	0.026148127	-0.047941969	-0.034810983	0.677728711	86.45%	0.9349277	4.639710518	2.392380371	-4.3863726	-3.18497437	1.75%	22.78%
0.016	5	0.81448698	0.003719041	0.002107252	0.047476796	0.024182183	-0.053754977	-0.03909573	0.757135168	84.86%	0.9383911	4.852757514	2.471739511	-5.4944708	-3.99610149	2.13%	28.42%
s [m]	C [kNm/(m*rad)]	R_c	α'_w	α'_v	$k_{x,cen}$	$k_{y,cen}$	$k_{x,ed} = G'$	$k_{y,ed} = F'$	q [kN/m^2]	r_{sym}	w [mm]	$\sigma_{x,cen}$ [MPa]	$\sigma_{y,cen}$ [MPa]	$\sigma_{x,ed}$ [MPa]	$\sigma_{y,ed}$ [MPa]	diff. w	diff. $\sigma_{x,cen}$
0.018	0.00000001	407243492	0.00772408	0.004911771	0.078419513	0.042579389	0	0	0.397865934	92.04%	1.0241486	4.212061141	2.287019928	0	0	0.00%	0.00%
0.018	0.1	40.7243492	0.007445228	0.004715915	0.076259051	0.041313221	-0.003667884	-0.002671159	0.412964808	91.74%	1.0246381	4.251461081	2.303222366	-0.2044855	-0.14891778	0.05%	0.94%
0.018	0.2	20.3621746	0.007193328	0.004539031	0.074307865	0.04016834	-0.006986949	-0.005086059	0.427621262	91.45%	1.0251056	4.289709122	2.318872888	-0.4033487	-0.29361245	0.09%	1.84%
0.018	0.5	8.14486984	0.006565156	0.004098131	0.069444304	0.03730808	-0.015290971	-0.011118763	0.469124037	90.62%	1.0263895	4.398028981	2.362785835	-0.9684039	-0.70417065	0.22%	4.42%
0.018	1	4.07243492	0.005809797	0.003568446	0.063601139	0.03385644	-0.025339679	-0.018403486	0.531050774	89.38%	1.0281974	4.559683605	2.427230947	-1.8166486	-1.31938005	0.40%	8.25%
0.018	2	2.03621746	0.004883634	0.002920006	0.056447186	0.029598842	-0.037790434	-0.027421823	0.633433407	87.33%	1.0309166	4.826997016	2.531100883	-3.2315926	-2.34493635	0.66%	14.60%
0.018	3.5	1.16355283	0.00413945	0.002400198	0.05071089	0.026148127	-0.047941969	-0.034810983	0.749265551	85.01%	1.0336129	5.129449584	2.644905205	-4.8493708	-3.52116052	0.92%	21.78%
0.018	5	0.81448698	0.003719041	0.002107252	0.047476796	0.024182183	-0.053754977	-0.03909573	0.835385293	83.29%	1.0353741	5.354291317	2.727194458	-6.0623258	-4.40909966	1.10%	27.12%
