# Supplementary material for: The Influence of Elastic Support of Component Glass Panes on Deflection and Stress in Insulating Glass Units—Analytical Model
Source: Materials (Basel). 2024 Sep 23;17(18):4669. doi: 10.3390/ma17184669 (PMC11433723; doi:10.3390/ma17184669)
Supplement: Supplementary file 1 [file materials-17-04669-s001.zip › File S6.pdf]

File S6
Calculations for Asymmetrically Loaded IGUs—Spreadsheet Printout.

Reference IGU parameters

Width (of IGU)	a =	60	cm
		0.6	m
Aspect ratio	ε =	1.5	
Glass pane thickness	d =	4	mm
		0.004	m
Gas cavity thickness	s =	16	mm
Young's modulus	E =	70	GPa
		70000000	kPa
Poisson's ratio	μ =	0.2	
Flexural rigidity	D =	0.38888889	kNm
Wind pressure	$q_{zi,n}$ =	0.3	kPa

"diff. w " means the percentage change in deflection at the pane center with respect to the case of simply support.

"diff. $\sigma_{x,cen}$" means percentage change in stress $\sigma_{x,cen}$ with respect to the case of simply support.

Other symbols are explained in the main text.

Data in green are presented in Figures 9–12 in the main text.

Table S6-1. The influence of rotational stiffness on static quantities in IGUs loaded with a wind pressure of 0.3 kN/m^2 for various widths of the unit **(data related to Figure 9 in main text)**.

a [m]	C [kNm/(m*rad)]	R_c	α'_w	α'_v	$k_{x,cen}$	$k_{y,cen}$	$k_{x,ed} = G'$	$k_{y,ed} = F'$	q [kN/m^2]	r_{win}	w [mm]	$\sigma_{x,cen}$ [MPa]	$\sigma_{y,cen}$ [MPa]	$\sigma_{x,ed}$ [MPa]	$\sigma_{y,ed}$ [MPa]	diff. w	diff. $\sigma_{x,cen}$
0.4	0.00000001	610865238	0.00772408	0.004911771	0.078419513	0.042579389	0	0	0.190579771	36.47%	0.096903	0.896710373	0.486886212	0	0	0.00%	0.00%
0.4	0.1	61.0865238	0.007534977	0.004778947	0.076954346	0.041720874	-0.002486672	-0.001811215	0.191396535	36.20%	0.094936	0.883727707	0.479113842	-0.02855642	-0.02079962	-2.03%	-1.45%
0.4	0.2	30.5432619	0.007358466	0.004654986	0.075586956	0.040919014	-0.004810428	-0.003502695	0.192189017	35.94%	0.093096	0.871618963	0.471851103	-0.05547068	-0.04039078	-3.93%	-2.80%
0.4	0.5	12.2173048	0.006893031	0.00432822	0.071982424	0.03880199	-0.010951517	-0.007967846	0.194431164	35.19%	0.088225	0.839737594	0.452658961	-0.12775897	-0.09295186	-8.96%	-6.35%
0.4	1	6.10865238	0.006280331	0.003898331	0.067240273	0.036008334	-0.019070937	-0.013860744	0.197771146	34.08%	0.081764	0.797891148	0.427284573	-0.22630086	-0.16447532	-15.62%	-11.02%
0.4	2	3.05432619	0.005437012	0.003307286	0.060720019	0.032146696	-0.03033114	-0.022018106	0.203277406	32.24%	0.072755	0.740580484	0.392081821	-0.36993813	-0.26854701	-24.92%	-17.41%
0.4	3.5	1.74532925	0.004672566	0.002772442	0.05481896	0.028623346	-0.040654017	-0.029499704	0.209481314	30.17%	0.064434	0.689012867	0.359763365	-0.51097541	-0.3707782	-33.51%	-23.16%
0.4	5	1.22173048	0.004201038	0.002443162	0.051185101	0.026435002	-0.047095586	-0.034191622	0.214074733	28.64%	0.059202	0.657446215	0.33954396	-0.60491851	-0.43917375	-38.91%	-26.68%
a [m]	C [kNm/(m*rad)]	R_c	α'_w	α'_v	$k_{x,cen}$	$k_{y,cen}$	$k_{x,ed} = G'$	$k_{y,ed} = F'$	q [kN/m^2]	r_{win}	w [mm]	$\sigma_{x,cen}$ [MPa]	$\sigma_{y,cen}$ [MPa]	$\sigma_{x,ed}$ [MPa]	$\sigma_{y,ed}$ [MPa]	diff. w	diff. $\sigma_{x,cen}$
0.6	0.00000001	407243492	0.00772408	0.004911771	0.078419513	0.042579389	0	0	0.160236532	46.59%	0.412466	1.696365559	0.921074436	0	0	0.00%	0.00%
0.6	0.1	40.7243492	0.007445228	0.004715915	0.076259051	0.041313221	-0.003667884	-0.002671159	0.160631559	46.46%	0.398555	1.653697383	0.895887961	-0.07953902	-0.05792478	-3.37%	-2.52%
0.6	0.2	20.3621746	0.007193328	0.004539031	0.074307865	0.04016834	-0.006986949	-0.005086059	0.161015467	46.33%	0.385991	1.615236615	0.873142744	-0.15187593	-0.11055611	-6.42%	-4.78%
0.6	0.5	8.14486984	0.006565156	0.004098131	0.069444304	0.03730808	-0.015290971	-0.011118763	0.162105015	45.96%	0.354667	1.519731452	0.816456637	-0.33463031	-0.24332497	-14.01%	-10.41%
0.6	1	4.07243492	0.005809797	0.003568446	0.063601139	0.03385644	-0.025339679	-0.018403486	0.163737413	45.42%	0.317021	1.405874603	0.748381388	-0.56012221	-0.40680079	-23.14%	-17.12%
0.6	2	2.03621746	0.004883634	0.002920006	0.056447186	0.029598842	-0.037790434	-0.027421823	0.166453697	44.52%	0.270904	1.268438779	0.665122954	-0.84919825	-0.61620262	-34.32%	-25.23%
0.6	3.5	1.16355283	0.00413945	0.002400198	0.05071089	0.026148127	-0.047941969	-0.034810983	0.169552918	43.48%	0.233898	1.160754211	0.598521304	-1.09737459	-0.79681101	-43.29%	-31.57%
0.6	5	0.81448698	0.003719041	0.002107252	0.047476796	0.024182183	-0.053754977	-0.03909573	0.171875016	42.71%	0.213021	1.101610138	0.56110228	-1.24728357	-0.90714319	-48.35%	-35.06%
a [m]	C [kNm/(m*rad)]	R_c	α'_w	α'_v	$k_{x,cen}$	$k_{y,cen}$	$k_{x,ed} = G'$	$k_{y,ed} = F'$	q [kN/m^2]	r_{win}	w [mm]	$\sigma_{x,cen}$ [MPa]	$\sigma_{y,cen}$ [MPa]	$\sigma_{x,ed}$ [MPa]	$\sigma_{y,ed}$ [MPa]	diff. w	diff. $\sigma_{x,cen}$
0.8	0.00000001	305432619	0.00772408	0.004911771	0.078419513	0.042579389	0	0	0.153397243	48.87%	1.247954	2.887040904	1.567574606	0	0	0.00%	0.00%
0.8	0.1	30.5432619	0.007358466	0.004654986	0.075586956	0.040919014	-0.004810428	-0.003502695	0.153580178	48.81%	1.190301	2.786077944	1.508243862	-0.17730872	-0.1291067	-4.62%	-3.50%
0.8	0.2	15.271631	0.007038495	0.004430329	0.073108783	0.039464067	-0.009029959	-0.006571467	0.153757182	48.75%	1.139855	2.697840111	1.456292093	-0.33322104	-0.24249848	-8.66%	-6.55%
0.8	0.5	6.10865238	0.006280331	0.003898331	0.067240273	0.036008334	-0.019070937	-0.013860744	0.154255385	48.58%	1.020369	2.489321793	1.333075071	-0.70603072	-0.51314267	-18.24%	-13.78%
0.8	1	3.05432619	0.005437012	0.003307286	0.060720019	0.032146696	-0.03033114	-0.022018106	0.154990587	48.34%	0.887565	2.258647552	1.195784474	-1.12824989	-0.8190238	-28.88%	-21.77%
0.8	2	1.5271631	0.004492399	0.00264656	0.053429861	0.027788774	-0.043107652	-0.031283225	0.156185183	47.94%	0.739014	2.002788606	1.04164674	-1.61586636	-1.1726343	-40.78%	-30.63%
0.8	3.5	0.87266463	0.003795878	0.002160745	0.048067467	0.024542565	-0.052687699	-0.038304015	0.157506311	47.50%	0.629716	1.817023059	0.927746136	-1.99167483	-1.44794976	-49.54%	-37.06%
0.8	5	0.61086524	0.003424422	0.001902366	0.045213845	0.022795296	-0.057868998	-0.042178631	0.15846831	47.18%	0.571563	1.719590807	0.866959694	-2.20089656	-1.60415434	-54.20%	-40.44%
a [m]	C [kNm/(m*rad)]	R_c	α'_w	α'_v	$k_{x,cen}$	$k_{y,cen}$	$k_{x,ed} = G'$	$k_{y,ed} = F'$	q [kN/m^2]	r_{win}	w [mm]	$\sigma_{x,cen}$ [MPa]	$\sigma_{y,cen}$ [MPa]	$\sigma_{x,ed}$ [MPa]	$\sigma_{y,ed}$ [MPa]	diff. w	diff. $\sigma_{x,cen}$
1.0	0.00000001	244346095	0.00772408	0.004911771	0.078419513	0.042579389	0	0	0.151410351	49.53%	3.0073	4.452572257	2.417610083	0	0	0.00%	0.00%
1.0	0.1	24.4346095	0.007274546	0.004596057	0.074936917	0.040537592	-0.005916193	-0.004307225	0.15150626	49.50%	2.834072	4.257529488	2.303137124	-0.33612759	-0.24471435	-5.76%	-4.38%
1.0	0.2	12.2173048	0.006893031	0.00432822	0.071982424	0.03880199	-0.010951517	-0.007967846	0.151598477	49.47%	2.687073	4.092159705	2.205870946	-0.6225875	-0.45296752	-10.65%	-8.09%
1.0	0.5	4.88692191	0.006030584	0.003723206	0.06530839	0.034866949	-0.022394193	-0.016269607	0.151855017	49.38%	2.354849	3.719027503	1.985520436	-1.27525144	-0.92648307	-21.70%	-16.47%
1.0	1	2.44346095	0.005134329	0.003095388	0.058382231	0.030754699	-0.034403074	-0.024966985	0.152225682	49.26%	2.009769	3.332728116	1.75562064	-1.96388675	-1.42523114	-33.17%	-25.15%
1.0	2	1.22173048	0.004201038	0.002443162	0.051185101	0.026435002	-0.047095586	-0.034191622	0.152808734	49.06%	1.650742	2.933073961	1.514812199	-2.69873136	-1.95929195	-45.11%	-34.13%
1.0	3.5	0.6981317	0.00355589	0.001993745	0.046223254	0.023415197	-0.056028869	-0.040792786	0.153427408	48.86%	1.402897	2.65946778	1.347199896	-3.22363657	-2.34702431	-53.35%	-40.27%
1.0	5	0.48869219	0.003225577	0.001764318	0.043688364	0.021854251	-0.060665761	-0.044312989	0.153861635	48.71%	1.276181	2.520736144	1.260949069	-3.50029997	-2.55677585	-57.56%	-43.39%

Table S6-2. The influence of rotational stiffness on static quantities in IGUs loaded with a wind pressure of 0.3 kN/m2 for various aspect ratios of the unit **(data related to Figure 10 in main text)**.

ε	C [kNm/(m*rad)]	R_c	α'_w	α'_v	$k_{x,cen}$	$k_{y,cen}$	$k_{x,ed} = G'$	$k_{y,ed} = F'$	q [kN/m^2]	r_{win}	w [mm]	$\sigma_{x,cen}$ [MPa]	$\sigma_{y,cen}$ [MPa]	$\sigma_{x,ed}$ [MPa]	$\sigma_{y,ed}$ [MPa]	diff. w	diff. $\sigma_{x,cen}$
1.0	0.00000001	407243492	0.00406241	0.001702503	0.044263846	0.044215019	0	0	0.168522275	43.83%	0.22815	1.007024932	1.005914096	0	0	0.00%	0.00%
1.0	0.1	40.7243492	0.003944625	0.001646959	0.043296025	0.043247198	-0.001993806	-0.001993806	0.169067596	43.64%	0.222252	0.988193903	0.987079473	-0.04550689	-0.04550689	-2.59%	-1.87%
1.0	0.2	20.3621746	0.003836424	0.001595939	0.042406887	0.04235806	-0.003828841	-0.003828841	0.169597582	43.47%	0.216833	0.970934243	0.969816319	-0.08766389	-0.08766389	-4.96%	-3.58%
1.0	0.5	8.14486984	0.003558883	0.001465093	0.040125883	0.040077056	-0.008553314	-0.008553314	0.171101785	42.97%	0.202931	0.92685738	0.925729541	-0.19757079	-0.19757079	-11.05%	-7.96%
1.0	1	4.07243492	0.003209807	0.001300583	0.037256134	0.037207308	-0.014540556	-0.014540556	0.17335564	42.21%	0.185437	0.871905741	0.870763045	-0.34029281	-0.34029281	-18.72%	-13.42%
1.0	2	2.03621746	0.002757237	0.001087444	0.033533533	0.033484707	-0.022411338	-0.022411338	0.177106675	40.96%	0.162738	0.801766703	0.800599281	-0.53584166	-0.53584166	-28.67%	-20.38%
1.0	3.5	1.16355283	0.002372438	0.000906432	0.030365498	0.030316671	-0.029255956	-0.029255956	0.181387828	39.54%	0.143411	0.743570787	0.742375146	-0.71640104	-0.71640104	-37.14%	-26.16%
1.0	5	0.81448698	0.002146139	0.000800125	0.028500386	0.028451559	-0.033384547	-0.033384547	0.184596837	38.47%	0.132027	0.710245949	0.709029155	-0.83196204	-0.83196204	-42.13%	-29.47%
ε	C [kNm/(m*rad)]	R_c	α'_w	α'_v	$k_{x,cen}$	$k_{y,cen}$	$k_{x,ed} = G'$	$k_{y,ed} = F'$	q [kN/m^2]	r_{win}	w [mm]	$\sigma_{x,cen}$ [MPa]	$\sigma_{y,cen}$ [MPa]	$\sigma_{x,ed}$ [MPa]	$\sigma_{y,ed}$ [MPa]	diff. w	diff. $\sigma_{x,cen}$
1.2	0.00000001	407243492	0.005650587	0.002848026	0.059305886	0.04485606	0	0	0.163766901	45.41%	0.308389	1.311166058	0.991701622	0	0	0.00%	0.00%
1.2	0.1	40.7243492	0.00547072	0.00274641	0.057888214	0.04374899	-0.002724864	-0.002351929	0.164227995	45.26%	0.299414	1.283426818	0.969949201	-0.06041236	-0.0521441	-2.91%	-2.12%
1.2	0.2	20.3621746	0.005306727	0.002653776	0.05659586	0.042739072	-0.005214832	-0.00449939	0.164676092	45.11%	0.29123	1.25819798	0.95014396	-0.11593235	-0.10002717	-5.56%	-4.04%
1.2	0.5	8.14486984	0.004891376	0.002419219	0.053323914	0.040178712	-0.011548354	-0.009953786	0.165947859	44.68%	0.270509	1.194613571	0.900122126	-0.25871733	-0.22299427	-12.28%	-8.89%
1.2	1	4.07243492	0.004379529	0.002130326	0.0492947	0.037017278	-0.019420483	-0.016716104	0.167853339	44.05%	0.244984	1.11702779	0.838818959	-0.44007205	-0.37879028	-20.56%	-14.81%
1.2	2	2.03621746	0.003732766	0.001765641	0.044209618	0.033008637	-0.029518894	-0.025362429	0.171024402	42.99%	0.212749	1.020724665	0.762113134	-0.68154091	-0.58557522	-31.01%	-22.15%
1.2	3.5	1.16355283	0.003197156	0.001464109	0.040005941	0.029671271	-0.038077058	-0.032672529	0.174643502	41.79%	0.186078	0.943214977	0.699555784	-0.89773795	-0.77031606	-39.66%	-28.06%
1.2	5	0.81448698	0.002888057	0.001290405	0.037584275	0.02773451	-0.043136699	-0.036997746	0.177356232	40.88%	0.170699	0.899883733	0.664049895	-1.03282591	-0.8858404	-44.65%	-31.37%
ε	C [kNm/(m*rad)]	R_c	α'_w	α'_v	$k_{x,cen}$	$k_{y,cen}$	$k_{x,ed} = G'$	$k_{y,ed} = F'$	q [kN/m^2]	r_{win}	w [mm]	$\sigma_{x,cen}$ [MPa]	$\sigma_{y,cen}$ [MPa]	$\sigma_{x,ed}$ [MPa]	$\sigma_{y,ed}$ [MPa]	diff. w	diff. $\sigma_{x,cen}$
1.5	0.00000001	407243492	0.00772408	0.004911771	0.078419513	0.042579389	0	0	0.160236532	46.59%	0.412466	1.696365559	0.921074436	0	0	0.00%	0.00%
1.5	0.1	40.7243492	0.007445228	0.004715915	0.076259051	0.041313221	-0.003667884	-0.002671159	0.160631559	46.46%	0.398555	1.653697383	0.895887961	-0.07953902	-0.05792478	-3.37%	-2.52%
1.5	0.2	20.3621746	0.007193328	0.004539031	0.074307865	0.04016834	-0.006986949	-0.005086059	0.161015467	46.33%	0.385991	1.615236615	0.873142744	-0.15187593	-0.11055611	-6.42%	-4.78%
1.5	0.5	8.14486984	0.006565156	0.004098131	0.069444304	0.03730808	-0.015290971	-0.011118763	0.162105015	45.96%	0.354667	1.519731452	0.816456637	-0.33463031	-0.24332497	-14.01%	-10.41%
1.5	1	4.07243492	0.005809797	0.003568446	0.063601139	0.03385644	-0.025339679	-0.018403486	0.163737413	45.42%	0.317021	1.405874603	0.748381388	-0.56012221	-0.40680079	-23.14%	-17.12%
1.5	2	2.03621746	0.004883634	0.002920006	0.056447186	0.029598842	-0.037790434	-0.027421823	0.166453697	44.52%	0.270904	1.268438779	0.665122954	-0.84919825	-0.61620262	-34.32%	-25.23%
1.5	3.5	1.16355283	0.00413945	0.002400198	0.05071089	0.026148127	-0.047941969	-0.034810983	0.169552918	43.48%	0.233898	1.160754211	0.598521304	-1.09737459	-0.79681101	-43.29%	-31.57%
1.5	5	0.81448698	0.003719041	0.002107252	0.047476796	0.024182183	-0.053754977	-0.03909573	0.171875016	42.71%	0.213021	1.101610138	0.56110228	-1.24728357	-0.90714319	-48.35%	-35.06%
ε	C [kNm/(m*rad)]	R_c	α'_w	α'_v	$k_{x,cen}$	$k_{y,cen}$	$k_{x,ed} = G'$	$k_{y,ed} = F'$	q [kN/m^2]	r_{win}	w [mm]	$\sigma_{x,cen}$ [MPa]	$\sigma_{y,cen}$ [MPa]	$\sigma_{x,ed}$ [MPa]	$\sigma_{y,ed}$ [MPa]	diff. w	diff. $\sigma_{x,cen}$
2.0	0.00000001	407243492	0.01012872	0.008808206	0.100002869	0.03671657	0	0	0.15774648	47.42%	0.532468	2.129638586	0.781907798	0	0	0.00%	0.00%
2.0	0.1	40.7243492	0.009699354	0.008408809	0.096660113	0.035307718	-0.004742796	-0.002877449	0.15809458	47.30%	0.511022	2.062994396	0.753564457	-0.1012244	-0.06141272	-4.03%	-3.13%
2.0	0.2	20.3621746	0.009315999	0.008052252	0.093675625	0.034049542	-0.008976684	-0.005452042	0.158432879	47.19%	0.491874	2.003575351	0.728266545	-0.19199725	-0.11661067	-7.62%	-5.92%
2.0	0.5	8.14486984	0.008378243	0.007180234	0.086375305	0.03097055	-0.019329562	-0.011777698	0.159392952	46.87%	0.445043	1.85862801	0.666425802	-0.41593445	-0.25343307	-16.42%	-12.73%
2.0	1	4.07243492	0.007284073	0.006163161	0.077857745	0.027375498	-0.031397187	-0.019230083	0.16083119	46.39%	0.390413	1.690463757	0.594382589	-0.68170233	-0.41752761	-26.68%	-20.62%
2.0	2	2.03621746	0.005990499	0.004961442	0.067788222	0.023121821	-0.045628361	-0.028214849	0.163223551	45.59%	0.325856	1.493725636	0.509493471	-1.00542913	-0.62171926	-38.80%	-29.86%
2.0	3.5	1.16355283	0.004988341	0.004031114	0.059986498	0.019825056	-0.056593394	-0.035425892	0.165951104	44.68%	0.275877	1.343901457	0.444148636	-1.26788439	-0.79366038	-48.19%	-36.90%
2.0	5	0.81448698	0.004436744	0.003519334	0.05569133	0.018011468	-0.062581858	-0.03956913	0.167992686	44.00%	0.24839	1.263024372	0.408482309	-1.41929474	-0.8973888	-53.35%	-40.69%

Table S6-3. The influence of rotational stiffness on static quantities in IGUs loaded with a wind pressure of 0.3 kN/m^2 for various thicknesses of glass panes **(data related to Figure 11 in main text)**.

d [m]	D [kNm]	C [kNm/(m*rad)]	R_c	α'_w	α'_v	$k_{x,cen}$	$k_{y,cen}$	$k_{x,ed} = G'$	$k_{y,ed} = F'$	q [kN/m^2]	r_{win}	w [mm]	$\sigma_{x,cen}$ [MPa]	$\sigma_{y,cen}$ [MPa]	$\sigma_{x,ed}$ [MPa]	$\sigma_{y,ed}$ [MPa]	diff. w	diff. $\sigma_{x,cen}$
0.004	0.38888889	0.00000001	407243492	0.00772408	0.004911771	0.078419513	0.042579389	0	0	0.160236532	46.59%	0.412466	1.696365559	0.921074436	0	0	0.00%	0.00%
0.004	0.38888889	0.1	40.7243492	0.007445228	0.004715915	0.076259051	0.041313221	-0.003667884	-0.002671159	0.160631559	46.46%	0.398555	1.653697383	0.895887961	-0.07953902	-0.05792478	-3.37%	-2.52%
0.004	0.38888889	0.2	20.3621746	0.007193328	0.004539031	0.074307865	0.04016834	-0.006986949	-0.005086059	0.161015467	46.33%	0.385991	1.615236615	0.873142744	-0.15187593	-0.11055611	-6.42%	-4.78%
0.004	0.38888889	0.5	8.14486984	0.006565156	0.004098131	0.069444304	0.03730808	-0.015290971	-0.011118763	0.162105015	45.96%	0.354667	1.519731452	0.816456637	-0.33463031	-0.24332497	-14.01%	-10.41%
0.004	0.38888889	1	4.07243492	0.005809797	0.003568446	0.063601139	0.03385644	-0.025339679	-0.018403486	0.163737413	45.42%	0.317021	1.405874603	0.748381388	-0.56012221	-0.40680079	-23.14%	-17.12%
0.004	0.38888889	2	2.03621746	0.004883634	0.002920006	0.056447186	0.029598842	-0.037790434	-0.027421823	0.166453697	44.52%	0.270904	1.268438779	0.665122954	-0.84919825	-0.61620262	-34.32%	-25.23%
0.004	0.38888889	3.5	1.16355283	0.00413945	0.002400198	0.05071089	0.026148127	-0.047941969	-0.034810983	0.169552918	43.48%	0.233898	1.160754211	0.598521304	-1.09737459	-0.79681101	-43.29%	-31.57%
0.004	0.38888889	5	0.81448698	0.003719041	0.002107252	0.047476796	0.024182183	-0.053754977	-0.03909573	0.171875016	42.71%	0.213021	1.101610138	0.56110228	-1.24728357	-0.90714319	-48.35%	-35.06%
d [m]	D [kNm]	C [kNm/(m*rad)]	R_c	α'_w	α'_v	$k_{x,cen}$	$k_{y,cen}$	$k_{x,ed} = G'$	$k_{y,ed} = F'$	q [kN/m^2]	r_{win}	w [mm]	$\sigma_{x,cen}$ [MPa]	$\sigma_{y,cen}$ [MPa]	$\sigma_{x,ed}$ [MPa]	$\sigma_{y,ed}$ [MPa]	diff. w	diff. $\sigma_{x,cen}$
0.006	1.3125	0.00000001	1374446786	0.00772408	0.004911771	0.078419513	0.042579389	0	0	0.17973284	40.09%	0.137082	0.845673709	0.459174869	0	0	0.00%	0.00%
0.006	1.3125	0.1	137.444679	0.007638394	0.004851584	0.077755595	0.042190451	-0.001126394	-0.000820577	0.180027905	39.99%	0.135784	0.839890613	0.455727507	-0.01216694	-0.00886361	-0.95%	-0.68%
0.006	1.3125	0.2	68.7223393	0.007555342	0.00479325	0.077112127	0.041813359	-0.002218728	-0.00161611	0.180319524	39.89%	0.134525	0.834289328	0.452385903	-0.0240048	-0.01748497	-1.87%	-1.35%
0.006	1.3125	0.5	27.4889357	0.007320826	0.004628554	0.075295392	0.040747952	-0.005306311	-0.003863519	0.181174298	39.61%	0.130967	0.818495382	0.442948899	-0.05768204	-0.04199822	-4.46%	-3.21%
0.006	1.3125	1	13.7444679	0.006972739	0.004384169	0.072599601	0.039164832	-0.009898316	-0.007202581	0.182535349	39.15%	0.125677	0.795119615	0.428937979	-0.10840755	-0.07888354	-8.32%	-5.98%
0.006	1.3125	2	6.87223393	0.006402165	0.003983787	0.068182951	0.036564545	-0.017452803	-0.012687242	0.18504182	38.32%	0.116978	0.757001837	0.405958192	-0.1937699	-0.14086022	-14.67%	-10.49%
0.006	1.3125	3.5	3.92699082	0.005764165	0.003536468	0.063248361	0.033647401	-0.025949406	-0.018845101	0.188341855	37.22%	0.107199	0.714738816	0.380232836	-0.29324156	-0.21295928	-21.80%	-15.48%
0.006	1.3125	5	2.74889357	0.005295075	0.003207902	0.059623577	0.031494402	-0.032238295	-0.023399118	0.191191137	36.27%	0.099964	0.683969969	0.361287037	-0.36982058	-0.26842224	-27.08%	-19.12%
d [m]	D [kNm]	C [kNm/(m*rad)]	R_c	α'_w	α'_v	$k_{x,cen}$	$k_{y,cen}$	$k_{x,ed} = G'$	$k_{y,ed} = F'$	q [kN/m^2]	r_{win}	w [mm]	$\sigma_{x,cen}$ [MPa]	$\sigma_{y,cen}$ [MPa]	$\sigma_{x,ed}$ [MPa]	$\sigma_{y,ed}$ [MPa]	diff. w	diff. $\sigma_{x,cen}$
0.008	3.11111111	0.00000001	3257947937	0.00772408	0.004911771	0.078419513	0.042579389	0	0	0.20542907	31.52%	0.066099	0.543700609	0.295212744	0	0	0.00%	0.00%
0.008	3.11111111	0.1	325.794794	0.007687601	0.004886148	0.078136867	0.042413825	-0.000479454	-0.000349311	0.205612018	31.46%	0.065846	0.54222341	0.294326735	-0.00332713	-0.00242401	-0.38%	-0.27%
0.008	3.11111111	0.2	162.897397	0.007651605	0.004860864	0.07785796	0.042250427	-0.000952681	-0.000694043	0.205793733	31.40%	0.065596	0.540765455	0.293451969	-0.00661688	-0.0048205	-0.76%	-0.54%
0.008	3.11111111	0.5	65.1589587	0.007546413	0.004786979	0.077042949	0.04177281	-0.002336202	-0.001701651	0.206331612	31.22%	0.064863	0.536503357	0.290892981	-0.01626859	-0.01184977	-1.87%	-1.32%
0.008	3.11111111	1	32.5794794	0.007379885	0.004670027	0.075752868	0.041016342	-0.004528311	-0.003297396	0.207204542	30.93%	0.0637	0.52975142	0.286833566	-0.03166718	-0.0230592	-3.63%	-2.57%
0.008	3.11111111	2	16.2897397	0.007076291	0.004456862	0.073401467	0.039636024	-0.008531037	-0.006208791	0.208867211	30.38%	0.061569	0.517426641	0.279404972	-0.06013757	-0.04376744	-6.85%	-4.83%
0.008	3.11111111	3.5	9.30842268	0.006682562	0.004180511	0.070353049	0.03784329	-0.013735707	-0.009989865	0.211172208	29.61%	0.058785	0.501410544	0.269711474	-0.09789523	-0.07119839	-11.07%	-7.78%
0.008	3.11111111	5	6.51589587	0.006347974	0.003945775	0.067763631	0.03631719	-0.018172308	-0.013209093	0.213276734	28.91%	0.056399	0.487768699	0.261414391	-0.1308059	-0.09508023	-14.68%	-10.29%
d [m]	D [kNm]	C [kNm/(m*rad)]	R_c	α'_w	α'_v	$k_{x,cen}$	$k_{y,cen}$	$k_{x,ed} = G'$	$k_{y,ed} = F'$	q [kN/m^2]	r_{win}	w [mm]	$\sigma_{x,cen}$ [MPa]	$\sigma_{y,cen}$ [MPa]	$\sigma_{x,ed}$ [MPa]	$\sigma_{y,ed}$ [MPa]	diff. w	diff. $\sigma_{x,cen}$
0.01	6.07638889	0.00000001	6363179565	0.00772408	0.004911771	0.078419513	0.042579389	0	0	0.230067848	23.31%	0.037902	0.389703068	0.211596805	0	0	0.00%	0.00%
0.01	6.07638889	0.1	636.317956	0.007705342	0.00489861	0.078274326	0.042494347	-0.000246266	-0.000179425	0.23016802	23.28%	0.037827	0.389150929	0.211266138	-0.00122434	-0.00089204	-0.20%	-0.14%
0.01	6.07638889	0.2	318.158978	0.007686732	0.004885537	0.078130129	0.042409878	-0.000490884	-0.000357638	0.230267771	23.24%	0.037752	0.388602375	0.210937567	-0.00244155	-0.00177882	-0.40%	-0.28%
0.01	6.07638889	0.5	127.263591	0.007631654	0.00484685	0.077703378	0.042159855	-0.001215013	-0.000885126	0.230564422	23.15%	0.037529	0.386977701	0.20996415	-0.006051	-0.0044081	-0.98%	-0.70%
0.01	6.07638889	1	63.6317956	0.00754229	0.004784083	0.077011007	0.041754088	-0.002390444	-0.001741148	0.231050409	22.98%	0.037168	0.384337975	0.208381659	-0.01192996	-0.00868953	-1.94%	-1.38%
0.01	6.07638889	2	31.8158978	0.007372153	0.004664598	0.07569298	0.040981211	-0.00463014	-0.0033715	0.231991936	22.67%	0.036478	0.379299476	0.205357907	-0.02320175	-0.01689467	-3.76%	-2.67%
0.01	6.07638889	3.5	18.180513	0.007136453	0.004499099	0.073867379	0.039909687	-0.00773715	-0.0056316	0.233332759	22.22%	0.035515	0.372290674	0.201144329	-0.03899514	-0.02838319	-6.30%	-4.47%
0.01	6.07638889	5	12.7263591	0.006921415	0.004348143	0.072202198	0.038931214	-0.010576392	-0.007695301	0.234594568	21.80%	0.034632	0.365866058	0.197273909	-0.05359314	-0.03899396	-8.63%	-6.12%

Table S6-4. The influence of rotational stiffness on static quantities in IGUs loaded with a wind pressure of 0.3 kN/m^2 for various thicknesses of gas cavity **(data related to Figure 12 in main text)**.

s [m]	C [kNm/(m*rad)]	R_c	α'_w	α'_v	$k_{x,cen}$	$k_{y,cen}$	$k_{x,ed} = G'$	$k_{y,ed} = F'$	q [kN/m^2]	r_{win}	w [mm]	$\sigma_{x,cen}$ [MPa]	$\sigma_{y,cen}$ [MPa]	$\sigma_{x,ed}$ [MPa]	$\sigma_{y,ed}$ [MPa]	diff. w	diff. $\sigma_{x,cen}$
0.012	0.00000001	407243492	0.00772408	0.004911771	0.078419513	0.042579389	0	0	0.157810536	47.40%	0.406221	1.670682428	0.907129284	0	0	0.00%	0.00%
0.012	0.1	40.7243492	0.007445228	0.004715915	0.076259051	0.041313221	-0.003667884	-0.002671159	0.158117377	47.29%	0.392317	1.627813949	0.881865651	-0.07829409	-0.05701815	-3.42%	-2.57%
0.012	0.2	20.3621746	0.007193328	0.004539031	0.074307865	0.04016834	-0.006986949	-0.005086059	0.158415975	47.19%	0.379759	1.58915965	0.859046411	-0.14942399	-0.10877126	-6.51%	-4.88%
0.012	0.5	8.14486984	0.006565156	0.004098131	0.069444304	0.03730808	-0.015290971	-0.011118763	0.159265533	46.91%	0.348455	1.493111357	0.802155325	-0.32876882	-0.23906282	-14.22%	-10.63%
0.012	1	4.07243492	0.005809797	0.003568446	0.063601139	0.03385644	-0.025339679	-0.018403486	0.160544274	46.49%	0.310839	1.378457825	0.733786767	-0.54919894	-0.39886753	-23.48%	-17.49%
0.012	2	2.03621746	0.004883634	0.002920006	0.056447186	0.029598842	-0.037790434	-0.027421823	0.162687928	45.77%	0.264776	1.239742228	0.650075531	-0.8299864	-0.60226195	-34.82%	-25.79%
0.012	3.5	1.16355283	0.00413945	0.002400198	0.05071089	0.026148127	-0.047941969	-0.034810983	0.165158283	44.95%	0.227836	1.130668676	0.583008258	-1.06893179	-0.7761585	-43.91%	-32.32%
0.012	5	0.81448698	0.003719041	0.002107252	0.047476796	0.024182183	-0.053754977	-0.03909573	0.167026553	44.32%	0.207012	1.070534554	0.545274011	-1.21209865	-0.88155337	-49.04%	-35.92%
s [m]	C [kNm/(m*rad)]	R_c	α'_w	α'_v	$k_{x,cen}$	$k_{y,cen}$	$k_{x,ed} = G'$	$k_{y,ed} = F'$	q [kN/m^2]	r_{win}	w [mm]	$\sigma_{x,cen}$ [MPa]	$\sigma_{y,cen}$ [MPa]	$\sigma_{x,ed}$ [MPa]	$\sigma_{y,ed}$ [MPa]	diff. w	diff. $\sigma_{x,cen}$
0.014	0.00000001	407243492	0.00772408	0.004911771	0.078419513	0.042579389	0	0	0.159033961	46.99%	0.40937	1.683634388	0.914161801	0	0	0.00%	0.00%
0.014	0.1	40.7243492	0.007445228	0.004715915	0.076259051	0.041313221	-0.003667884	-0.002671159	0.159385696	46.87%	0.395464	1.640871254	0.888939426	-0.07892212	-0.05747551	-3.40%	-2.54%
0.014	0.2	20.3621746	0.007193328	0.004539031	0.074307865	0.04016834	-0.006986949	-0.005086059	0.159727753	46.76%	0.382904	1.602318829	0.866159823	-0.15066131	-0.10967195	-6.47%	-4.83%
0.014	0.5	8.14486984	0.006565156	0.004098131	0.069444304	0.03730808	-0.015290971	-0.011118763	0.16069973	46.43%	0.351593	1.506556929	0.80937879	-0.3317294	-0.24121559	-14.11%	-10.52%
0.014	1	4.07243492	0.005809797	0.003568446	0.063601139	0.03385644	-0.025339679	-0.018403486	0.162159318	45.95%	0.313966	1.392324839	0.741168517	-0.55472378	-0.40288006	-23.31%	-17.30%
0.014	2	2.03621746	0.004883634	0.002920006	0.056447186	0.029598842	-0.037790434	-0.027421823	0.164596969	45.13%	0.267883	1.254289826	0.65770376	-0.83972577	-0.60932911	-34.56%	-25.50%
0.014	3.5	1.16355283	0.00413945	0.002400198	0.05071089	0.026148127	-0.047941969	-0.034810983	0.167391972	44.20%	0.230917	1.145960439	0.590893171	-1.08338859	-0.78665568	-43.59%	-31.94%
0.014	5	0.81448698	0.003719041	0.002107252	0.047476796	0.024182183	-0.053754977	-0.03909573	0.169495767	43.50%	0.210073	1.086360657	0.553334996	-1.23001754	-0.89458569	-48.68%	-35.48%
s [m]	C [kNm/(m*rad)]	R_c	α'_w	α'_v	$k_{x,cen}$	$k_{y,cen}$	$k_{x,ed} = G'$	$k_{y,ed} = F'$	q [kN/m^2]	r_{win}	w [mm]	$\sigma_{x,cen}$ [MPa]	$\sigma_{y,cen}$ [MPa]	$\sigma_{x,ed}$ [MPa]	$\sigma_{y,ed}$ [MPa]	diff. w	diff. $\sigma_{x,cen}$
0.016	0.00000001	407243492	0.00772408	0.004911771	0.078419513	0.042579389	0	0	0.160236532	46.59%	0.412466	1.696365559	0.921074436	0	0	0.00%	0.00%
0.016	0.1	40.7243492	0.007445228	0.004715915	0.076259051	0.041313221	-0.003667884	-0.002671159	0.160631559	46.46%	0.398555	1.653697383	0.895887961	-0.07953902	-0.05792478	-3.37%	-2.52%
0.016	0.2	20.3621746	0.007193328	0.004539031	0.074307865	0.04016834	-0.006986949	-0.005086059	0.161015467	46.33%	0.385991	1.615236615	0.873142744	-0.15187593	-0.11055611	-6.42%	-4.78%
0.016	0.5	8.14486984	0.006565156	0.004098131	0.069444304	0.03730808	-0.015290971	-0.011118763	0.162105015	45.96%	0.354667	1.519731452	0.816456637	-0.33463031	-0.24332497	-14.01%	-10.41%
0.016	1	4.07243492	0.005809797	0.003568446	0.063601139	0.03385644	-0.025339679	-0.018403486	0.163737413	45.42%	0.317021	1.405874603	0.748381388	-0.56012221	-0.40680079	-23.14%	-17.12%
0.016	2	2.03621746	0.004883634	0.002920006	0.056447186	0.029598842	-0.037790434	-0.027421823	0.166453697	44.52%	0.270904	1.268438779	0.665122954	-0.84919825	-0.61620262	-34.32%	-25.23%
0.016	3.5	1.16355283	0.00413945	0.002400198	0.05071089	0.026148127	-0.047941969	-0.034810983	0.169552918	43.48%	0.233898	1.160754211	0.598521304	-1.09737459	-0.79681101	-43.29%	-31.57%
0.016	5	0.81448698	0.003719041	0.002107252	0.047476796	0.024182183	-0.053754977	-0.03909573	0.171875016	42.71%	0.213021	1.101610138	0.56110228	-1.24728357	-0.90714319	-48.35%	-35.06%
s [m]	C [kNm/(m*rad)]	R_c	α'_w	α'_v	$k_{x,cen}$	$k_{y,cen}$	$k_{x,ed} = G'$	$k_{y,ed} = F'$	q [kN/m^2]	r_{win}	w [mm]	$\sigma_{x,cen}$ [MPa]	$\sigma_{y,cen}$ [MPa]	$\sigma_{x,ed}$ [MPa]	$\sigma_{y,ed}$ [MPa]	diff. w	diff. $\sigma_{x,cen}$
0.018	0.00000001	407243492	0.00772408	0.004911771	0.078419513	0.042579389	0	0	0.161418775	46.19%	0.415509	1.708881533	0.927870226	0	0	0.00%	0.00%
0.018	0.1	40.7243492	0.007445228	0.004715915	0.076259051	0.041313221	-0.003667884	-0.002671159	0.161855558	46.05%	0.401592	1.666298417	0.902714551	-0.0801451	-0.05836616	-3.35%	-2.49%
0.018	0.2	20.3621746	0.007193328	0.004539031	0.074307865	0.04016834	-0.006986949	-0.005086059	0.162279774	45.91%	0.389022	1.627919582	0.879998731	-0.15306847	-0.11142421	-6.37%	-4.74%
0.018	0.5	8.14486984	0.006565156	0.004098131	0.069444304	0.03730808	-0.015290971	-0.011118763	0.163482253	45.51%	0.35768	1.532643034	0.823393222	-0.33747331	-0.24539225	-13.92%	-10.31%
0.018	1	4.07243492	0.005809797	0.003568446	0.063601139	0.03385644	-0.025339679	-0.018403486	0.165279811	44.91%	0.320008	1.419117872	0.755431103	-0.56539854	-0.41063283	-22.98%	-16.96%
0.018	2	2.03621746	0.004883634	0.002920006	0.056447186	0.029598842	-0.037790434	-0.027421823	0.168260231	43.91%	0.273845	1.282205237	0.672341582	-0.85841466	-0.62289031	-34.09%	-24.97%
0.018	3.5	1.16355283	0.00413945	0.002400198	0.05071089	0.026148127	-0.047941969	-0.034810983	0.171644615	42.79%	0.236784	1.175073909	0.60590499	-1.1109124	-0.8066409	-43.01%	-31.24%
0.018	5	0.81448698	0.003719041	0.002107252	0.047476796	0.024182183	-0.053754977	-0.03909573	0.174169126	41.94%	0.215865	1.116313927	0.568591617	-1.26393174	-0.91925132	-48.05%	-34.68%
